# Gas reactions under intrapore condensation regime within tailored metal–organic framework catalysts

**DOI:** 10.1038/s41467-019-10013-6

**Published:** 2019-05-06

**Authors:** Iker Agirrezabal-Telleria, Ignacio Luz, Manuel A. Ortuño, Mikel Oregui-Bengoechea, Iñaki Gandarias, Núria López, Marty A. Lail, Mustapha Soukri

**Affiliations:** 1 Department of Chemical and Environmental Engineering, Engineering School of the University of the Basque Country (UPV/EHU), Plaza Torres Quevedo 1, 48013 Bilbao, Spain; 2 RTI International, 3040 E Cornwallis Road, Research Triangle Park, NC 27709 USA; 3 Institute of Chemical Research of Catalonia (ICIQ), The Barcelona Institute of Science and Technology (BIST), Av. Països Catalans 16, 43007 Tarragona, Spain

**Keywords:** Catalytic mechanisms, Heterogeneous catalysis, Metal-organic frameworks

## Abstract

Production of 1-butene, a major monomer in polymer industry, is dominated by homogeneous protocols via ethylene dimerization. Homogeneous catalysts can achieve high selectivity but require large amounts of activators and solvents, and exhibit poor recyclability; in turn, heterogeneous systems are robust but lack selectivity. Here we show how the precise engineering of metal–organic frameworks (MOFs) holds promise for a sustainable process. The key to the (Ru)HKUST-1 MOF activity is the intrapore reactant condensation that enhances ethylene dimerization with high selectivity (> 99% 1-butene) and high stability (> 120 h) in the absence of activators and solvents. According to spectroscopy, kinetics, and modeling, the engineering of defective nodes via controlled thermal approaches rules the activity, while intrapore ethylene condensation accounts for selectivity and stability. The combination of well-defined actives sites with the concentration effect arising from condensation regimes paves the way toward the development of robust MOF catalysts for diverse gas-phase reactions.

## Abstract (web summary)

The search for robust heterogeneous catalysts for the production of linear α-olefins is still a challenge as they do not show well-defined sites and lack the high selectivity reported for homogeneous counterparts. Here the authors show how the precise engineering of metal–organic frameworks (MOFs) holds promise for a sustainable process.

## Introduction

Linear α-olefins, derived from light alkene oligomerization reactions, are broadly used as lubricants and co-monomers in polymer synthesis, and represent one of few examples for which homogeneous catalysts^1^ are still employed in bulk chemical production. Transition-metal compounds selectively convert light alkenes into industrially-relevant products^2^, but their limited recyclability and excessive use of solvent and activators present environmental and economic limitations. The search for robust heterogeneous catalysts is still a challenge as they do not show well-defined sites and lack the high selectivity reported for homogeneous counterparts.

Metal–organic frameworks (MOFs)^3^ hold promise to get the best ingredients from both worlds, i.e., selective and well-defined catalytic sites distributed on porous solids^4–6^. While typical pristine MOFs are hardly reactive during ethylene dimerization, recent advances in defect-engineering synthetic methods enable tailoring a broad range of lattice defects that enhance catalytic activity^7–11^. However, there are several challenges to render these materials into practice in large-scale processes. For instance, remarkable ethylene dimerization turnovers were obtained using Ni-containing MOFs^12–20^, but they still require large amounts of activators (up to 500 equivalents per active metal) to generate the active species. Moreover, the Ni-MOF catalysts undergo fast deactivation^12^. Given the narrow channels in MOFs, we propose the use of intrapore reactant condensation^21^ to provide catalyst stability during gas-phase reactions at mild temperatures.

Here, we present heterogeneous catalysis and defect synthesis methods for (Ru)HKUST-1 that allow controlled defect engineering and present unique activity, selectivity, and stability in ethylene dimerization. The thermal engineering of defects yields catalytically active species with remarkable activity in the absence of activators, high selectivity to 1-butene (>99% selectivity), and long-time stability (>120 h) under intrapore ethylene condensation regime. We prove that active Ru–H sites created through economic and versatile thermal approaches behave the same as those prepared via conventional ligand-engineered methods. Kinetic, spectroscopic, and theoretical methods provide new insights on the nature and concentration of catalytic sites and demonstrate the kinetic consequences of intrapore reactant condensation on the stability of MOF catalysts. The exquisite control over well-defined active sites combined with intrapore liquid regime opens new research opportunities to develop heterogeneous porous systems that work at wider temperature ranges than those under liquid phase.

## Results

### Ligand-engineered defective MOF catalysts

**Fig. 1:**
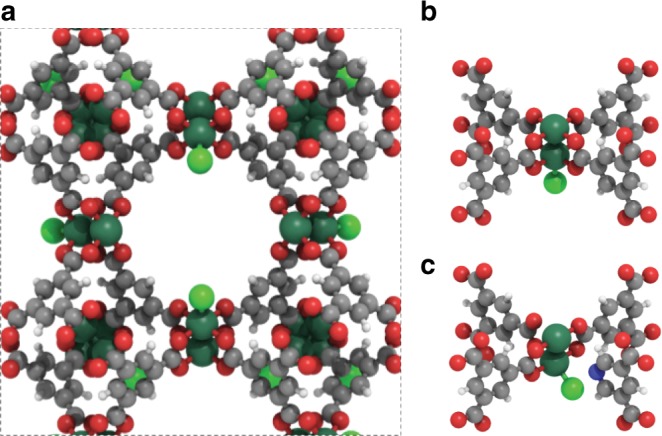
Structure of (Ru)HKUST-1 metal–organic framework. **a** Periodic structure of pristine (Ru)HKUST-1 with a pore size of 1.5 nm. **b** Paddle-wheel node of pristine (Ru)HKUST-1. **c** Paddle-wheel node of defective (Ru)HKUST-1. (Ru = dark green, Cl = green, O = red, N = blue, C = grey, H = white)

In order to tailor an active and stable MOF catalyst for ethylene dimerization, we chose the (Ru)-HKUST-1 material, which is amenable to include lattice defective sites without compromising the framework structure (Fig. 1)^7,22^. Previously, defective (Ru)HKUST-1, prepared via controlled in-synthesis incorporation of coordinatively deficient pyridine-3,5-dicarboxylate ligands, led to the formation of robust metal–hydride species upon H_2_ exposure at 150 °C^23,24^. Inspired by these results, we propose a new experimental set up where such defective MOFs can efficiently catalyze ethylene dimerization in the absence of activators.

**Fig. 2:**
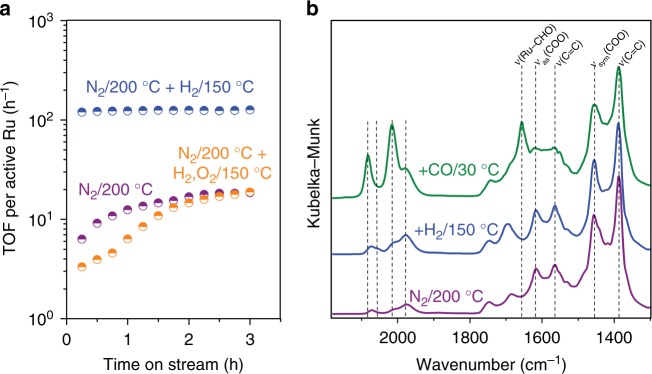
Catalytic activity and formation of the Ru–H active site in MOF_L25_. **a** Ethylene dimerization TOFs at 50 °C and 4.2 MPa ethylene after activation of MOF_L25_ in N_2_ at 200 °C, in N_2_ at 200 °C + H_2_ at 150 °C, and in N_2_ at 200 °C + H_2_ at 150 °C + O_2_ at 150 °C. Active Ru corresponds to one metal atom per defective node. **b** IR spectra after subsequent treatments for MOF_L25_: N_2_ at 200 °C, H_2_ at 150 °C, and CO at 30 °C. All spectra are shown after N_2_ flushing

Here we use direct catalytic evidences to learn on ethylene dimerization site requirements in (Ru)HKUST-1 MOFs containing 25% of pyridine-3,5-dicarboxylate linkers, namely MOF_L25_. Figure 2a shows ethylene dimerization turnover frequencies (TOFs) vs. time on stream at 50 °C using different pre-treated MOF_L25_. As discussed later, we consider one active Ru atom per defective node to calculate TOF values. The reaction pressure (4.2 MPa) and temperature (50 °C) conditions in Fig. 2a lead to the remarkable stabilization of active species, facilitated by the intrapore condensation of ethylene reactants within MOF_L25_, as discussed in next sections. After N_2_ treatment at 200 °C to remove labile adsorbates (purple line), MOF_L25_ exhibits dimerization activity after an induction period with 99% selectivity to 1-butene (Fig. 2a). Slow induction periods reflect the limited capacity of ethylene to form active Ru–H species at dimerization conditions (50 °C). Interestingly, further exposure to H_2_ at 150 °C (blue line) produces a catalyst with initial rates one order of magnitude higher than those found previously^24^, while maintaining high selectivity (Supplementary Fig. 7). The catalytic differences in Fig. 2a are attributed to the facile formation of Ru–H species in ligand-engineered open metal sites under H_2_ atmosphere at 150 °C (*vide infra*). The generation of Ru–H species is optimized at 150 °C in H_2_ without involving the degradation of the porous framework (see N_2_ physisorption in Supplementary Fig. 2). Higher pre-treatment temperatures (200 °C in H_2_) lead to complete MOF degradation into metallic Ru particles (Supplementary Fig. 4). The catalytic role of Ru–H species is supported by quenching under oxidizing environments (O_2_ at 150 °C, orange line in Fig. 2a), leading to inactive Ru species and induction periods in contact with ethylene. The results in Fig. 2a show compelling evidences on the role of Ru–H species in ligand-engineered defective MOFs to achieve stable alkene dimerization turnovers in the absence of co-catalysts and solvents.

To evaluate the (Ru)HKUST-1 pre-treatment effects in ethylene dimerization (Fig. 2a), we turn to IR spectroscopy. Figure 2b shows *in-situ* IR spectroscopic data of MOF_L25_ after successive gas pre-treatments. The two bands at 2059 and 1978 cm^−1^ after N_2_ and H_2_ (purple and blue lines) are characteristic of Ru–H species, as reported in literature^23^. Periodic density functional theory (DFT) calculations evidence the facile formation of Ru–H species via a heterolytic H–H bond breaking process under H_2_ atmosphere (Supplementary Fig. 27)^25–27^. After CO exposure (green line), MOF_L25_ exhibits two additional intense stretching vibrations at 2082 and 2015 cm^−1^, which correspond to single carbonyl species bonded to Ru^2^^+^ and Ru^+δ^. The latter were previously identified as partially reduced Ru (0 < δ < 2)^23,28^. Moreover, a C = O stretching band appears at 1656 cm^−1^, which is assigned to Ru(II)–formyl species^29^ coming from CO reduction via Ru–H. Even after N_2_ purging at 30 °C, these CO-adsorbed species are stable, suggesting the remarkable binding strength of CO to Ru species. At 30 °C, IR spectra rule out the presence of either sym-/asymmetric Ru(CO)_2_ gem-dicarbonyl or Ru_2_(CO) bridged-carbonyl species, as compared to −80 °C^23,30^. DFT-computed C = O frequencies of 1950–2050 cm^−1^ for CO and 1600–1700 cm^−1^ for formyl are in line with experimental data (Supplementary Fig. 28). MOF_L25_ treated at 200 °C in N_2_ also shows the bands that correspond to Ru–H species, and their presence is also confirmed by the initial dimerization activity and the induction period in contact with ethylene at 50 °C (Fig. 2a). These evidences suggest that active Ru–H species within the defects of (Ru)HKUST-1 can also be formed via thermal approaches in contact with N_2_ as discussed in the next section.

### Thermal-engineered defective MOF catalysts

**Fig. 3:**
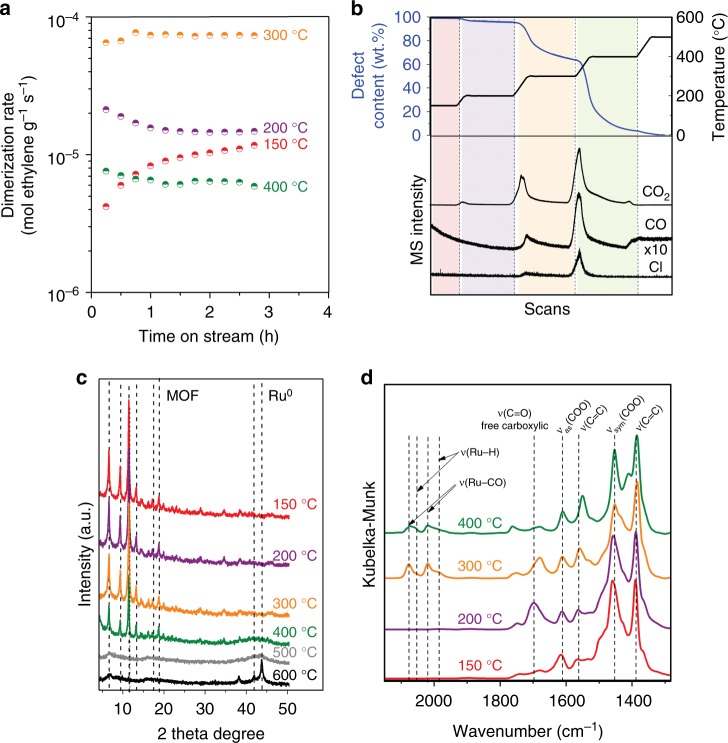
Thermal treatment effects on activity, surface species, and structure of MOF_L0_. **a** Ethylene dimerization rates at 50 °C and 4.2 MPa ethylene after MOF_L0_ thermal pre-treatment. **b** Thermogravimetric analysis (TGA) coupled to mass-spectrometry (MS) for MOF_L0_. **c** XRD data for MOF_L0_ in N_2_. **d** DRIFT spectra of MOF_L0_ in N_2_

Based on the above-mentioned catalytic and spectroscopic evidences for MOF_L25_ (Fig. 2), together with a potential partial decarboxylation of linkers at high temperature^31^, we next examine the generation of defects and Ru–H sites via a controlled thermal approach. We start from pristine (Ru)HKUST-1 without any pyridine-3,5-dicarboxylate linkers, namely MOF_L0_, and evaluate thermal pre-activation effects on ethylene dimerization activity (Fig. 3a). MOF_L0_ treated at 150 °C in N_2_ shows an induction period in contact with ethylene at 50 °C and 4.2 MPa. After thermal pre-activation at 200 °C in N_2_, MOF_L0_ exhibits greater initial rates than when treated at 150 °C, while maintaining selectivity. Temperature effects on ethylene dimerization activity are optimized for MOF_L0_ pre-treated at 300 °C, as the initial rates abruptly decrease when MOF_L0_ is pre-treated at 400 °C in N_2_. Such catalytic differences inspired us to perform analogous pre-treatment thermal experiments on MOF_L0_ via in-situ TGA-MS, XRD, and IR. TGA analyses (Fig. 3b) quantify the number of generated defects (blue line), XRD determines the MOF structural stability (Fig. 3c), and IR experiments (Fig. 3d) evaluate the nature of the resulting defective site as a function of temperature. The proposed mechanism for the thermal generation of defects is supported by MS (Fig. 3b), which identifies the composition of released species.

N_2_-treatments up to 200 °C incorporate only *ca*. 5% of defects in MOF_L0_ (purple area, Fig. 3b) and induce carboxylate protonation, as indicated by the significant appearance of a new band in IR at 1698 cm^−1^ (purple line, Fig. 3d), assigned to the stretching of free carboxylic acids. Heating from 200 to 300 °C increases the defect concentration up to 38% on MOF_L0_ (orange area, Fig. 3b). In this range, a progressive attenuation of IR signals occurs for both symmetric carboxylates [*ν*_sym_(COO)] and free carboxylic acids due to further release of CO_2_, while signals attributed to phenyl rings [ν(C = C)] remain constant (orange line, Fig. 3d). Moreover, intense IR signals appear between 2100–1900 cm^−1^, which are assigned to Ru–CO and Ru–H stretching bands. Similar bands were observed in MOF_L25_ at 200 °C (Fig. 2b), which contains a combination of ligand-engineered (25.0%) and thermal-engineered (8.2%) defects (Supplementary Fig. 13). The presence of Ru–CO and Ru–H species at defective Ru sites is supported by the simultaneous release of CO_2_ and CO at 300 °C (Fig. 3b), which can originate from the catalytic rupture of formic acid^32^. These evidences suggest a consequent reduction of Ru atoms with temperature to compensate the charge over the MOF node, which is consistent with the release of HCl (Fig. 3b) and elemental analysis (Supplementary Table 2). A similar metal reduction via dehalogenation was reported for Fe-containing MOFs^33^. XPS data (Supplementary Fig. 20) are also consistent with the reduction of Ru atoms and the dehalogenation process when MOF_L0_ is treated above 200 °C in N_2_. Nevertheless, in-situ XRD data indicate the remarkable structural stability of the thermal-engineered MOF_L0_ structure and the absence of Ru nanoparticles up to 300 °C (orange line, Fig. 3c).

N_2_-treatments at 400 °C (green area, Fig. 3b) lead to nearly complete MOF decarboxylation (up to 95%) and the consequent reduction of Ru–CO and Ru–H stretching bands (green line, Fig. 3d). These effects are consistent with the sintering of metallic Ru nanoclusters into larger crystalline domains at 400 °C, as supported by the appearance of a broad signal centered at 42 theta degree in XRD (green line, Fig. 3c). XPS data also support the advanced degree of Ru reduction (Supplementary Table 3) at 400 °C, which leads to the fusion of phenyl rings into a carbonaceous matrix^31^.

The differences between the number of engineered defects and active Ru–H species explain the activity trends observed in Fig. 3a as a function of thermal pre-treatment in N_2_. Compared to the more limited *in-synthesis* ligand engineering procedures, the thermal approach reveals a general and straightforward route to create active sites from *as-synthesized* MOF_L0_ without H_2_ activation. The active sites are the same in both cases but the thermal treatment, when properly controlled, is a much more appealing technique to design defect sites. Next, we will compare this versatile procedure to conventional ligand engineering approaches as a function of defect content.

### Role of defects in selective ethylene dimerization

In order to evaluate the influence of the type and number of defects on catalytic activity, we prepared and tested a series of ligand- and thermal-engineered MOFs. For ligand-engineered samples (% of defects): MOF_L10_ (20.4%), MOF_L25_ (33.3%), and MOF_L50_ (51.0%). For thermal-engineered samples (% of defects): MOF_L0_−200 (5.0%), MOF_L0_−250 (13.4%), MOF_L0_−300 (38%), MOF_L0_−350 (71.4%), and MOF_L0_−400 (95.0%).

**Fig. 4:**
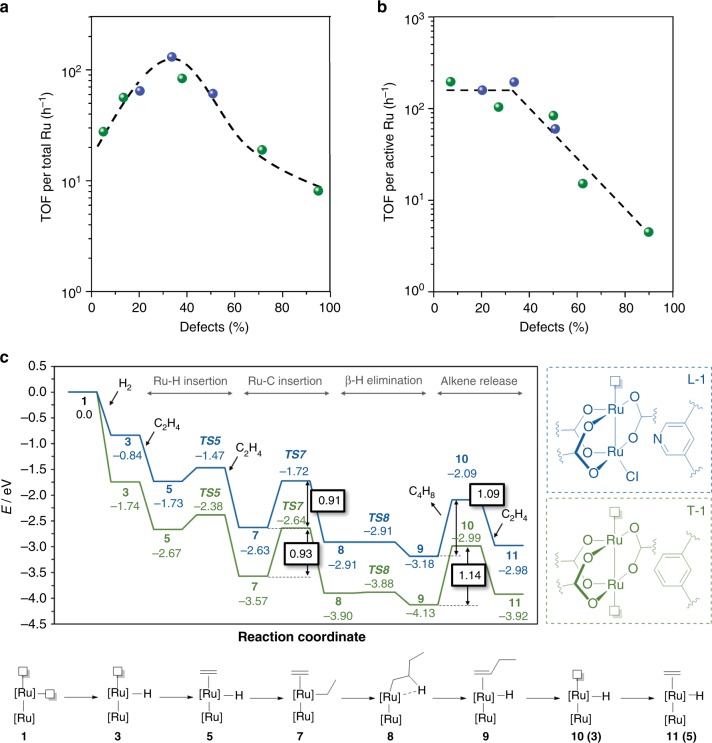
Effects of ligand- and thermal-engineered defects on catalytic activity. **a** Turnover frequencies per total Ru as a function of measured defects via ligand (blue) and thermal (green) approaches. **b** Turnover frequencies per active Ru as a function of measured defects via ligand (blue) and thermal (green) approaches. **c** Computed reaction mechanism and energy profile for ethylene dimerization via ligand- (**L**, blue) and thermal-engineered (**T**, green) defective MOFs

Figure 4a, b plot ethylene dimerization TOFs per total Ru in the MOF and per active Ru sites, respectively, as a function of measured defect contents for ligand- (blue) and thermal-engineered (green) MOFs. Figure 4a shows a volcano shape with a maximum TOF regardless of the method employed to create defects. The increase in activity parallels the higher concentration of active sites (the active Ru/total Ru fraction increases), but there is a maximum to the incorporation of active sites without compromising the mechanical stability. When defects are above 40%, partial amorphization for ligand-engineered samples (S_BET_ decreased from 974 m^2^/g for MOF_L25_ to 668 m^2^/g for MOF_L50_, Supplementary Fig. 1) causes smaller dimerization TOFs. Such decrease in TOFs for thermal-engineered catalysts is also attributed to the aggregation of Ru into larger inactive particles (Fig. 3c). This latter hypothesis is confirmed by testing the catalytic activity of Ru nanoparticles embedded on a carbonaceous matrix prepared at 500 °C under N_2_ (Supplementary Fig. 24), which show no ethylene dimerization products. Figure 4b displays a plateau at low defect content, suggesting that the nature of active sites in both ligand- and thermal-engineered MOFs is the same and that they behave as single ensemble catalysts. In-situ FTIR data indicate the presence of bound ethyl intermediates when MOF catalysts are exposed to ethylene (Supplementary Fig. 10). The optimized MOF_L25_ catalyst presents a TOF of *ca*. 200 h^−1^, which is comparable to related gas-phase systems under flow conditions: 16^14^, 252^13^, and 1570^12^ h^−1^ (Supplementary Table 4).

To get insights on the reaction mechanism and selectivity to dimers, we turn to computational modeling at periodic DFT level (see Computational Details). We use a unit cell of *ca*. 26 × 26 × 26 Å^3^, which prevents a spurious concentration of defect sites when computing periodic images. Benchmark calculations indicate that PBE-D2 is suitable to describe the electronic structure of (Ru)HKUST-1 (Supplementary Table 5). We computed the ethylene dimerization via a Cossee–Arlman mechanism^15,34,35^ using a ligand-engineered defective node. Figure 4c shows the model **L-1** used in the simulations (Supplementary Fig. 26) and the reaction profile in blue. From the active Ru–H species **3**, ethylene binds to Ru forming **5** with an adsorption energy of 0.89 eV. Species **5** undergoes insertion via **TS5** with a relative energy barrier of 0.26 eV. The resulting ethyl species binds a second ethylene as in **7**, which inserts into the Ru–C bond via **TS7** with an energy barrier of 0.91 eV. Such C–C bond formation appears to be the rate-determining step, where ethyl intermediates are potential resting states of the catalytic cycle in line with in-situ FTIR (Supplementary Fig. 10). The butyl intermediate (**8**) quickly undergoes β-H elimination through a barrierless process via **TS8** to form 1-butene (**9**). Direct desorption of 1-butene via **10** takes only 1.09 eV; alternatively, at high ethylene pressures, ethylene can directly substitute 1-butene from **9** to **11**^36^. To evaluate entropic contributions, we estimate the relative Gibbs energies for the C–C bond formation step, **7** to **TS7**, and the alkene desorption process, **9**–**10**. The resulting Gibbs (electronic) energy barriers are 0.91 (0.91) and 0.68 (1.09) eV, respectively, which further supports the C–C bond formation as the rate-determining step of the catalytic cycle. We also studied the reaction mechanism using a thermal-engineered defective node. Figure 4c shows the model used in the calculations **T-1** (Supplementary Fig. 26) and the reaction profile in green. Similar energies were observed, with a barrier of 0.93 eV (cf. 0.91 eV) for the C–C bond-forming step and 1.14 eV (cf. 1.09 eV) for alkene desorption. The kinetic resemblance between ligand- and thermal-engineered active sites toward dimerization is in line with previous experimental results (Fig. 4a, b).

The dimerization mechanism in Fig. 4c takes place on one metal atom, which allows defining TOFs per Ru atom per defective node (Figs. 2a and 4b). To further explore the role of the second metal, we prepared a periodic structure with a node containing only one Ru atom (Supplementary Fig. 30) and computed the desorption of 1-butene. Interestingly, we found a significantly higher value of 1.74 eV, compared to 1.09 eV for the bimetallic node. It seems that the second metal plays a key electronic role favoring the alkene desorption. Bader analyses located positive charges of +0.95 |e^−^| on Ru in the bimetallic model and +1.22 |e^−^| on Ru in the monometallic model. The higher charge of the latter is in line with the more energy-demanding desorption step. To further highlight the unique nature of the bimetallic active site, we attached mononuclear Ru and Ni species to bipyridine linkers in (Zr)UiO-67-(Bpy)^37,38^. However, under our activator-free conditions, no dimerization products were experimentally observed. These results explain how the active site controls the dimerization selectivity via: (i) the slower rate of ethylene insertion into Ru–alkyl groups (*ca*. 0.9 eV via **TS7**) compared to chain termination (barrierless step via **TS8**)^15^ and (ii) facile alkene desorption (*ca*. 1.1 eV from **9** to **10**). The first one is due to the engineered defect (via ligand or thermal approaches), and the second one arises from the bimetallic nature of the MOF node. Such facile desorption is further enhanced in the presence of a solvating environment, as shown below.

### Stability of MOF catalysts under intrapore condensation

**Fig. 5:**
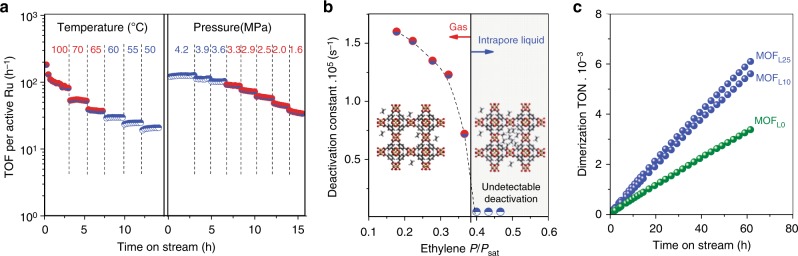
Stability of MOF catalysts. **a** Turnover frequencies per active Ru as a function of temperature at 4.2 MPa ethylene and pressure at 50 °C. **b** First-order deactivation constants, derived from ethylene pressure changes in (**a**), as a function of ethylene relative saturation pressure (*P/P*_*sat*_) within MOF_L25_. **c** Long-term stability during ethylene dimerization at 4.2 MPa ethylene and 50 °C for MOF_L25_, MOF_L10_, and MOF_L0_

After characterization and reactivity studies, we next evaluate the stability of these catalysts during ethylene dimerization with respect to reaction conditions. Figure 5a shows ethylene dimerization TOFs for H_2_-activated MOF_L25_ as a function of systematic changes in temperature (at constant 4.2 MPa) and ethylene pressure (at constant 50 °C). Stable TOFs are achieved at temperatures below 60 °C (at 4.2 MPa) or pressures above 3.3 MPa (at 50 °C). However, high temperatures (>60 °C, 4.2 MPa) or low pressures (<3.6 MPa, 50 °C) lead to fast deactivation rates. Such deactivation occurs via the irreversible binding of oligomer products in secondary reactions of primary dimer products^21^, as evidenced by the curved shape of ethylene dimerization rate data at high ethylene conversion (100 °C in Fig. 5a).

Figure 5b shows first-order deactivation constants (*k*_d_) as a function of ethylene relative saturation pressures (*P/P*_*sat*_). Deactivation is accurately measured by *k*_d_ within small changes in conversion for MOF_L25_. Figure 5b shows a continuous increase in *k*_d_ values below *P/P*_*sat*_ of 0.4, whereas deactivation is undetectable at *P/P*_*sat*_ above 0.4. Such reaction conditions and the narrow micropores of MOF_L25_ (1.5 nm diameter) suggest that active site stabilization is related to the intrapore condensation of ethylene molecules. We use N_2_ uptakes as surrogate to estimate ethylene intrapore condensation at reaction conditions (Supplementary Fig. 3)^21^. These analyses indicate that kinetically-relevant pores are liquid-filled at *P/P*_*sat*_ above 0.4. Condensation within MOF pores also leads to very high selectivity for C_4_ among products (>99%) and 1-butene (primary C_4_ product, Supplementary Fig. 6), as liquid ethylene promotes the desorption of bound alkenes by substitution and prevents the formation of larger oligomers. To support such claim, we estimated the Gibbs energy difference associated with the ligand exchange from **9** to **11** (Fig. 4c) using reported entropies in gas and liquid phases^39^. The electronic energy difference to exchange 1-butene by ethylene is endothermic by 0.20 eV. The resulting Gibbs energy differences in low-pressure (ethylene in gas phase) and high-pressure (ethylene in liquid phase to model intrapore condensation) are +0.01 and −0.33 eV, respectively. The exoergicity of the process only under intrapore condensation conditions further proves the benefits of our experimental protocol.

Although MOF_L25_ starts to deactivate at *P/P*_*sat*_ below 0.4, it can be regenerated in H_2_ at 150 °C (Supplementary Fig. 7). Such mild conditions are more feasible than high temperatures and oxidizing environments used in typical catalyst regeneration procedures. This suggests that the regeneration mechanism proceeds through Ru-catalyzed hydrogenation of bound alkenes rather than oligomer-desorption pathways.

The long-term stable rates, enabled by controlled intrapore ethylene condensation, indicate that the crystalline and porous structure of MOFs is maintained during catalytic conditions or regeneration steps. Such enhanced stability is independent of the synthetic protocol to create catalytic sites. Figure 5c shows turnover numbers (TONs) under intrapore liquid ethylene regime for ligand- (MOF_L25_ and MOF_L10_ H_2_-activated at 150 °C) and thermal-engineered (MOF_L0_ pre-treated at 300 °C in N_2_) catalysts, with values of 13,000. Our TON results outstand over reported Ru-based MOF catalysts^24^ and are comparable to the 16,000 reported for state-of-the-art Ni-MOF catalysts^12^. Both ligand and thermal approaches lead to materials with remarkable stability, showing a mean life of one order of magnitude larger than Ni-MOF systems requiring co-catalysts or organic solvents^12^.

In summary, we have developed a method for tailoring MOF defects via thermal-based protocols, resulting in defective nodes that behave as well-defined active sites for ethylene dimerization with remarkable activity (TOF of *ca*. 200 h^−1^) and high selectivity (99% to 1-butene). Moreover, we demonstrate that working under reactant intrapore condensation regime within thermal-engineered defective MOFs provides outstanding stability (>120 h) to these catalytic systems (TON of 13,000). Such enhanced stability represents a significant advancement in the field of synthesis and performance of porous catalysts for ethylene dimerization reactions. Due to the absence of co-catalysts and solvents, such intrapore condensation effects in narrow-pore MOFs open exciting opportunities for the design of competitive procedures in terms of process intensification, safety, and production costs in other reactions involving gaseous reactants and structured catalysts.

## Methods

### MOF synthesis

(Ru)HKUST-1 containing 0% (MOF_L0_), 10% (MOF_L10_), 25% (MOF_L25_), and 50% (MOF_L50_) of ligand-engineered defects were prepared by combining the appropriate mixture of organic ligands 1,3,5-benzenetricarboxylic acid and pyridine-3,5-dicarboxylic acid with Ru precursor [Ru_2_(CH_3_COO)_4_Cl],under solvothermal conditions at 160 °C according to literature^23^. In a typical synthesis, 1 g of Ru precursor, 600 mg of the selected mixture of organic ligand, 5 mL of acetic acid and 25 mL of H_2_O were loaded into a 100-mL autoclave and heated at 160 °C for 24 h. The resulting powder was thoroughly washed with H_2_O in a filtration funnel and purified in a Soxhlet apparatus with MeOH. All the samples were evacuated at 150 °C under vacuum.

Ru precursor [Ru_2_(CH_3_COO)_4_Cl] was prepared by mixing 10 g of RuCl_3_·*x*H_2_O (40 wt.%Ru) and 12 g of LiCl(anhydrous), previously evacuated at 80 °C overnight in a vacuum oven, with 70 mL of acetic anhydride and 350 mL of glacial acetic acid. The mixture was stirred and refluxed for 2–4 days until the solution turned into reddish color. After cooling down, the Ru precursor was collected by filtration and was thoroughly washed with acetone.

### Ethylene dimerization reactivity tests

Ethylene dimerization rates to butene products were measured in a high-pressure tubular reactor integrated in a fully-automated lab-scale reaction unit. As-prepared MOF samples (50 mg) were diluted with inert SiO_2_ (1:20 mass, Davisil-62, Sigma-Aldrich). Activation tests on ligand-engineered MOF samples were carried out in a flow of inert (N_2_, 2.0 cm^3^ g^−1^ s^−1^, 99.999%, Air Liquide) and oxidizing (O_2_, 2.0 cm^3^ g^−1^ s^−1^) or reducing environment (H_2_, 99.9%, 2.0 cm^3^ g^−1^ s^−1^) depending on the defect formation temperature and conditions. Kinetic isotope effects were evaluated using D_2_ (99%, Air Liquide) as activator instead of H_2_. Thermal-engineered MOF samples were treated in N_2_ for 1 h at each reaction temperature. Prior to each catalytic test, the reactor was flushed with N_2_ before cooling to reaction temperature, initially pressurized in N_2_ until desired setpoint and ethylene (99.9%, Air Liquide) was introduced at 10–20 mol g^−1^ h^−1^. Temperatures were measured with a K-type thermocouple (K-type, Omega) and controlled electronically. Pressure was electronically controlled with a servometer. The concentrations of ethylene and products were measured by flame ionization detection after chromatography separation (Agilent 6890; HP-5 methyl silicone). First-order deactivation constants (*k*_d_) were measured from rate values (*r*) in a specific time interval (*t*):

Eq. 1 $$\frac{r}{{r_0}} = - {\mathrm{exp}}[k_{\rm{d}}\left( {t - t_0} \right)]$$

### N_2_ sorption isotherms

MOF samples were analyzed in a Micromeritics ASAP (Accelerated Surface Area and Porosimetry) 2020 System. Samples were weighted into tubes with seal frits and degassed under vacuum (<500 µm Hg) with heating. Samples were initially heated at 150 °C and held for 4 h, and finally cooled to room temperature and backfilled with N_2_. The samples were re-weighted before analysis. The analysis adsorptive was N_2_ at 77 K. A multi-point BET surface area was determined from 6 measurements at relative pressures (*P* / *P*_*sat*_) ranging from 0.05 to 0.30. Single point adsorption total pore volume was measured near saturation pressure (*P*_*sat*_ ≈ 770 mmHg). Adsorption average pore width was also calculated. Pore size distribution plot was determined by Horvath-Kawazoe method using the Cylinder Pore Geometry (Saito-Foley) with Cheng-Yang Correction.

### X-ray diffraction

XRD was used to study the crystalline structure of the MOF catalyst. XRD patterns were recorded using a Panalytical Empyrean X-ray diffractometer with Cu *K*α radiation (*λ* = 1.54778 Å). The samples were prepared by filling the holder with the dry powder. Crystalline phase stability was investigated using an XRK900 high temperature oven chamber. Sample was first heated in the chamber from 25 to 800 °C with a heating rate of 3 °C/min. Diffraction patterns were measured throughout the whole heat treatment using Cu Kα X-ray radiation with a wavelength of 1.5418 Å and a 2*θ* range of 4.5°–60°. Each pattern was measured for 4 min using a step size and count time of 2*θ* = 0.0263° and 147 s/step, respectively.

### Fourier Transform Infrared Spectroscopy

In-situ FTIR analyses were carried out in a diffuse reflectance cell (Harrick) using a Bruker Vertex V70 module equipped with a liquid-nitrogen cooled MCT detector. Samples were sieved into fine powders (<30 μm particle size) and treated using same MOF activation procedures as described in main text in N_2_ or H_2_. Temperature was controlled with a Harrick module. CO adsorption experiments were carried out using 1% CO in He between −80 and 30 °C, but given the remarkable binding strength of CO onto Ru–H species, the spectra after CO adsorption are shown at 30 °C. Ethylene was also introduced at 50 °C at same reaction conditions but at atmospheric pressure. All IR spectra are measured in N_2_ to avoid signal-disturbance for gas-phase H_2_ or CO.

### H_2_-temperature programmed reduction

H_2_-TPR measurements of MOF catalysts were performed using an AutoChem II 2920 reactor (Micromeritics) equipped with a built-in TCD detector, and with the reactor downstream connected to a benchtop quadrupole mass spectrometer (TA). To mimic the MOF activation procedures as described in manuscript in N_2_ or H_2_, 100 mg sample was first degassed at 200 °C for 1 h in He (50 mL/min), and then treated at 150 °C for 1 h with H_2_ (50 mL/min). The TCD calibration was performed with H_2_ concentrations varied from 0 to 100% in Ar and a background test with an empty reactor at the same reaction condition was performed to create a baseline.

### Thermogravimetric analyses (TGA) and mass spectrometry (MS)

TGA-MS analyses of the MOF catalysts were performed using a TA Q500 unit coupled to a benchtop quadrupole mass spectrometer (TA).

### XPS measurements

X-ray Photoelectron Spectroscopy (XPS) was performed using a monochromatized Al *K*α source (hν = 1486.6 eV), operated at 225 W, on a Kratos Axis Ultra DLD with a pass energy for narrow scan spectra of 20 eV, corresponding to an instrument resolution of ~600 meV. Survey spectra were collected with a pass energy of 80 eV. Spectral fitting was performed using Casa XPS analysis software. Spectral positions were corrected by shifting the primary C 1 s core level position to 285.0 eV, and curves were fitted with quasi-Voigt lines following Shirley background subtraction.

### Computational details

All calculations reported in the manuscript were performed at periodic DFT level using the Vienna Ab-initio Simulation Package (VASP)^40,41^. The PBE functional^42^ was used together with Grimme’s D2 dispersion scheme^43^ and modified parameters for transition metals^44^. Core electrons were described by projector augmented wave (PAW)^45^ and valence electrons in plane waves with a kinetic energy cutoff of 450 eV. The Brillouin zone was sampled at the Γ-point through the Monkhorst–Pack method^46^. Transition states were located with the climbing image nudged elastic band method^47^. The assessment of the minima and transition states was performed by diagonalizing the numerical Hessian matrix obtained by ±0.015 Å displacements. Selected cluster calculations were computed at the DFT level using Gaussian09 for benchmark purposes (Supplementary Table 5). All inputs and final structures can be found in the ioChem-BD repository^48,49^.

## Supplementary information

Supplementary Information

## Data Availability

The data that support this article and other findings are available from the corresponding authors upon request. The computational data can be freely accessed through the ioChem-BD repository (10.19061/iochem-bd-1-83).
