# Supplementary material for: Gas reactions under intrapore condensation regime within tailored metal–organic framework catalysts
Source: Nat Commun. 2019 May 6;10:2076. doi: 10.1038/s41467-019-10013-6 (PMC6502813; doi:10.1038/s41467-019-10013-6)
Supplement: Supplementary file 1 — Supplementary Information [file 41467_2019_10013_MOESM1_ESM.pdf]

Gas reactions under intrapore condensation regime within tailored Metal–Organic Framework catalysts

Agirrezabal-Telleria *et al.*

Supplementary Figures and Tables

a) b)

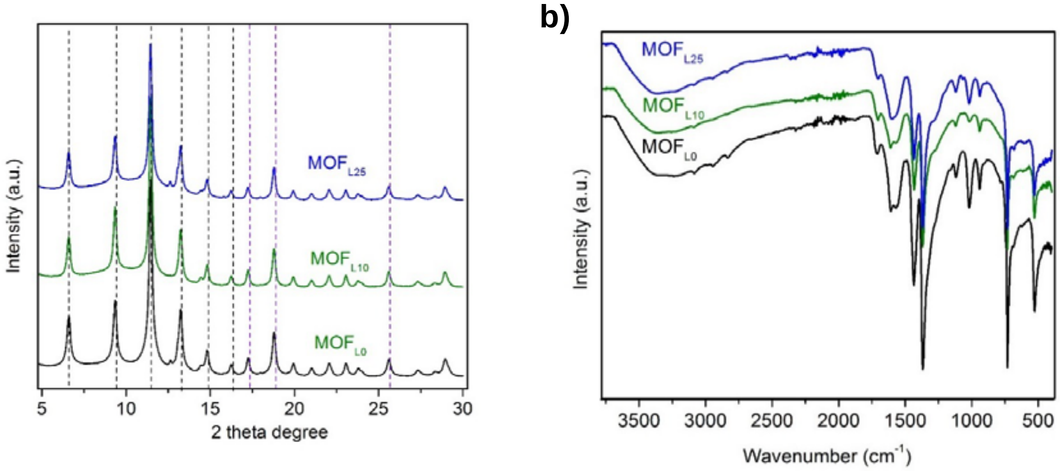

c)

Material	% N	% C	% H
**MOF$_{L0}$**	-	29.57	2.41
**MOF$_{L10}$**	0.39	30.21	2.47
**MOF$_{L25}$**	0.81	31.03	2.96

d)

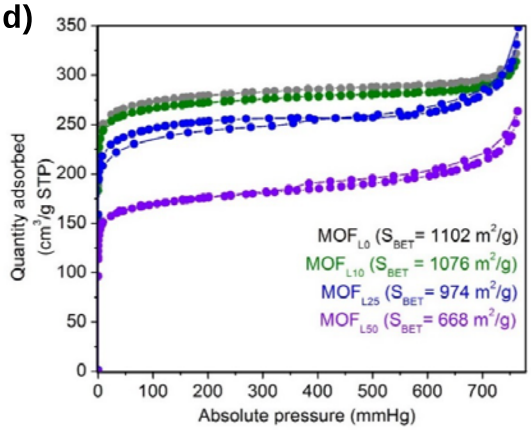

Supplementary Figure 1. (a) XRD, (b) FTIR, (c) CHN analysis and (d) N_2 sorption isotherms of as-synthesized MOF samples with 0%, 10% and 25% defect ligand-engineered compositions.

Supplementary Note 1. N_2 physisorption data of H_2-activated MOF catalysts.

Surface area of MOF catalysts was determined before and after H_2 activation at 150 °C to confirm the stability of the catalysts after forming the Ru–H active species. Pore diameter was determined to be 1.5 nm for both MOF catalysts after H_2 activation.

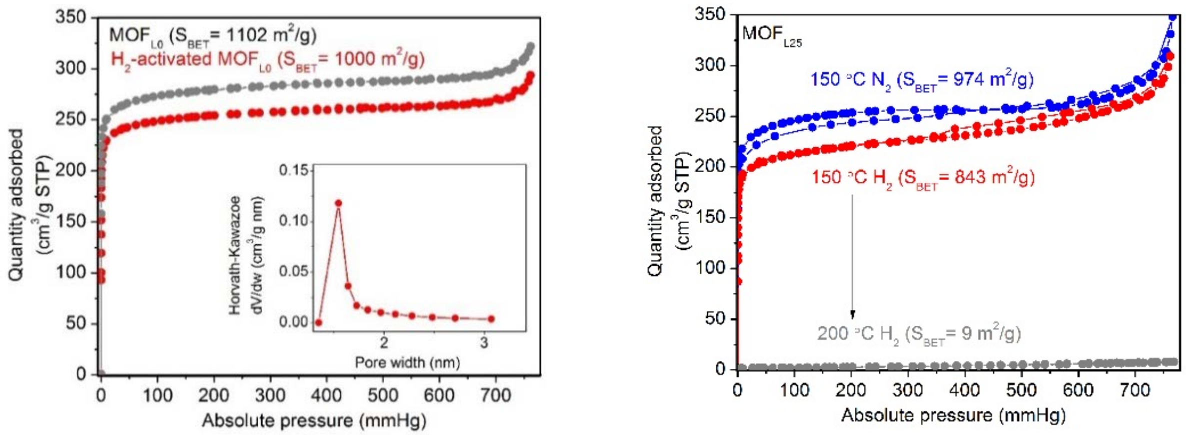

Supplementary Figure 2. N_2 sorption isotherms and pore volume distribution for 0 % (left) and 25 % (right) defect ligand-engineered MOF samples before and after activation under N_2 at 200 ºC + H_2 at 150 ºC and at 200 ºC.

Supplementary Note 2. Pore filling with N_2.

The physisorption data with N_2 (Supplementary Figure 3) are used as reference to estimate the required relative saturation pressure during ethylene dimerization. Inert gas molecules, such as N_2, condense into MOF micropores *via* capillary condensation following Kelvin's equation. In this work, N_2 is used as surrogate to predict the ethylene condensation during dimerization in MOF catalysts, as previously explained in Ni-based MCM-41 mesoporous materials.[1] As shown in the N_2 uptake data at 77 K (Supplementary Figure 3), the cumulative pore volume data for MOF catalysts show a plateau at ~ P/P_{sat} of 0.4. This P/P_{sat} is the one required to fill the pores below 1.5 nm (as shown in Supplementary Figure 2). This P/P_{sat} is used in Figure 5 (main text) to explain the deactivation effects in the presence of intrapore liquid-ethylene conditions.

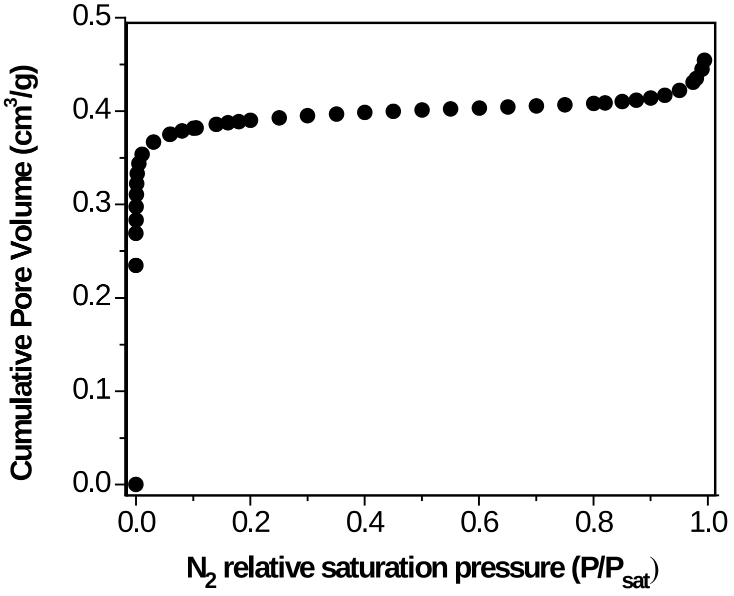

Supplementary Figure 3. Cumulative pore volume for 25 % defect ligand-engineered MOF samples as a function of N_2 relative pressure (P/P_{sat}).

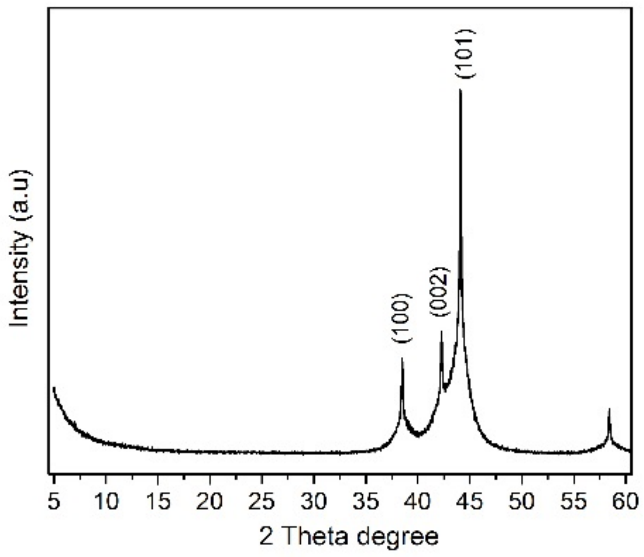

Supplementary Figure 4. XRD pattern for MOF_{L25} after treatment at 200 ºC in H_2.

Supplementary Note 3. TEM images after H_2 for ligand-engineered MOF catalysts.

Supplementary Figure 5 shows the TEM analysis for pristine MOF_{L0}, which leads to the formation of Ru nanoparticles when exposed to the beam at 80 kV. Pristine MOF_{L0}, however, only contains oxidized Ru atoms, suggesting that such TEM methods are not appropriate to evaluate the possible presence of metallic Ru.

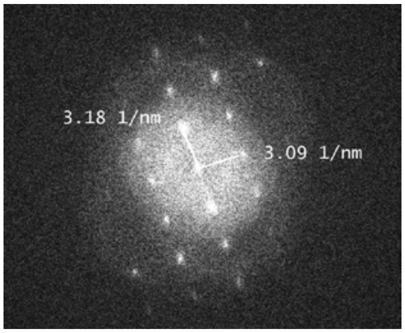

Supplementary Figure 5. TEM for pristine MOF_{L0} (without any thermal treatment). Measurement at 80 kV.

Supplementary Note 4. Product selectivity in the presence of intrapore liquid-ethylene.

Low reactant conversions allow the determination of primary butene products, as evidenced on $(Ni\text{-}OH)^+$.[1] Butenes (C_4) account for 100% products within defect-engineered MOF samples. As shown in Supplementary Figure 6, 1-butene selectivity within C_4 extrapolate to 100% at zero ethylene conversion, indicating that the primary ethylene-dimer product is 1-butene. This indicates the high-selectivity of such MOF structures for ethylene dimerization reactions, and also indicates the effects of intrapore liquids in reducing the formation of large oligomers that lead to deactivation.

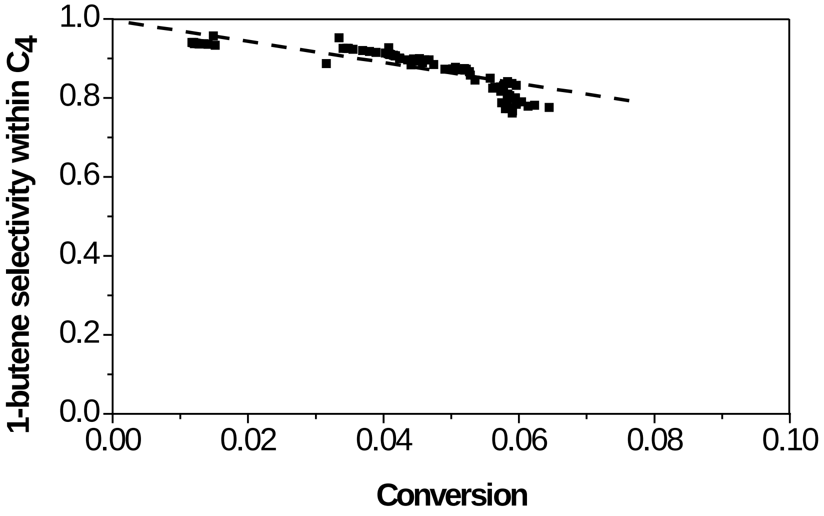

Supplementary Figure 6. 1-Butene selectivity as a function of ethylene conversion at 50 ºC and 4.2 MPa ethylene with 25% defect ligand-engineered MOF (MOF_{L25}).

Supplementary Note 5. Ru–H regeneration after deactivation.

Active-site regeneration in most oligomer-deactivated catalysts is usually carried out via post-reaction organic solvent extraction (determined by oligomer solubility) or high-temperature desorption (for weakly bound species) or calcination procedures (as chemical reaction to CO_2). Supplementary Figure 7 shows the dimerization rates *vs.* time-on-stream at 50 ºC for H_2-activated MOF_{L25}in the presence of intrapore liquids (4.2 MPa, blue dots) and under gaseous conditions (2.1 MPa, red dots). The presence of intrapore liquids leads to stable rates on time, and catalyst deactivates on time in the absence of liquid-ethylene. After this procedure, the reactor was flushed with N_2 at 150 ºC to remove physisorbed species and treated back in H_2 at 150 ºC, which allow to recover initial rates in the presence of intrapore liquids. These results indicate that deactivated Ru–H species regenerate via oligomer-hydrogenation rather than desorption, as 150 ºC treatments in N_2 does not produce similar effects.

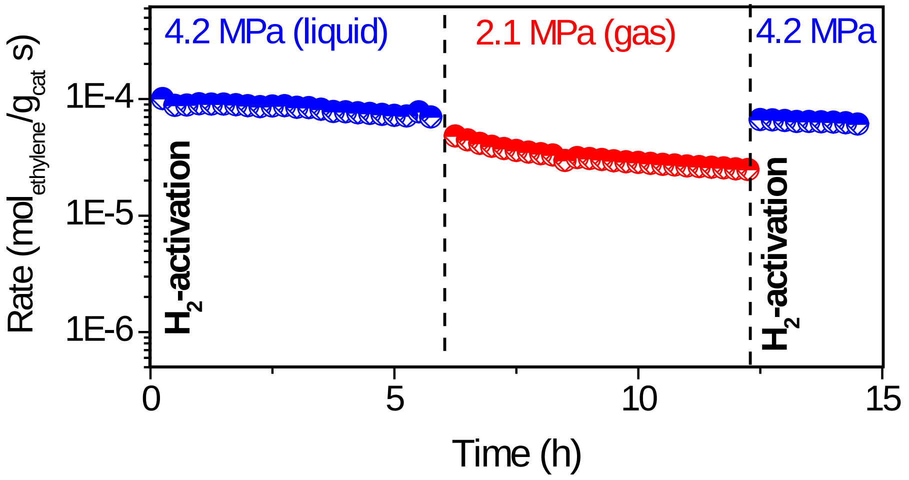

Supplementary Figure 7. Ethylene dimerization rates for 25% defect ligand-engineered MOF (MOF_{L25}) after activation under N_2 at 200 ºC + H_2 at 150 ºC in the presence of intrapore liquid (0-6 h), gas ethylene (6-12 h) and intrapore liquid after a second H_2activation at 150 ºC (12-15 h).

Supplementary Note 6. Experimental quantification of Ru–H formation energy with $H_2$for ligand-engineered MOF.

Supplementary Figure 8 shows the DRIFT spectra after H_2 treatments for MOF_{L25}. The increasing Ru–H peak (shown at 1970 cm^{-1}) is amplified in Supplementary Figure 8b. This confirms the catalytic activation of Ru species after H_2 treatment. The corrected integrated areas of Ru–H species are depicted in an Arrhenius plot, and the Ru–H activation energies (30.6 $kJmol^{-1}$, 0.32 eV) derived from the experimental IR data coincide with those obtained from DFT estimates (34.7 kJ mol^{-1}, 0.36 eV), as shown in Supplementary Figure 2 in the manuscript.

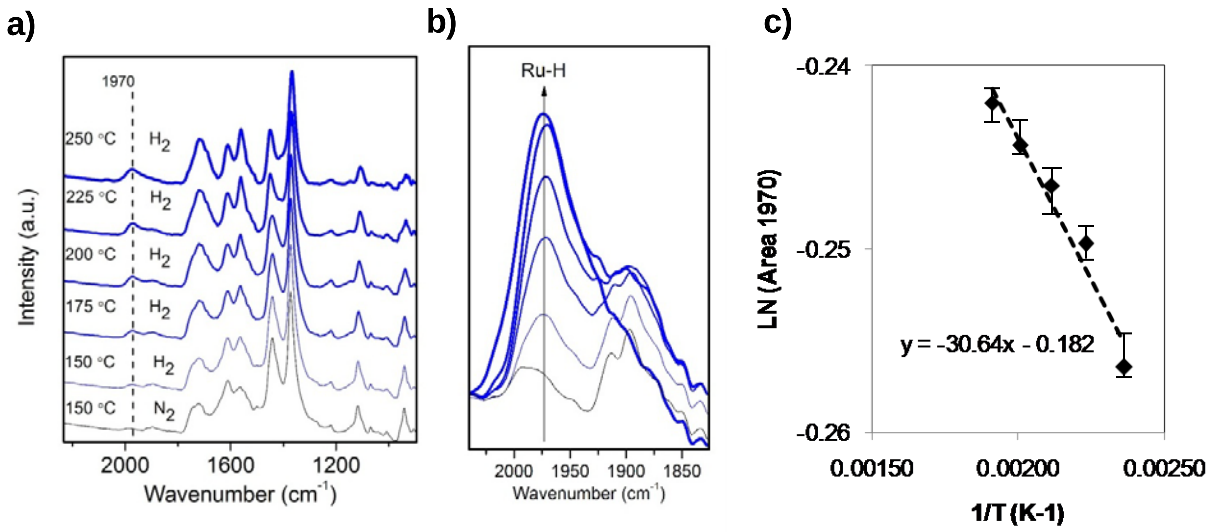

Supplementary Figure 8. a) DRIFT spectra after H_2 treatments at increasing temperatures for MOF_{L25}. b) DRIFT spectra in the Ru–H region after H_2 treatments at increasing temperatures for MOF_{L25}. c) Arrhenius plot of corrected activation constants (derived from integrated areas) as a function of inverse of reaction temperature (right), shown with error bars (standard deviation). The trend slope corresponds to Ru–H activation energy in kJ mol^{-1}.

Supplementary Note 7. Defect concentration on MOF_{L25} compared to MOF_{L0} evidenced by CO-DRIFT.

DRIFT analysis of CO probe molecules strongly adsorbed over the available catalytically active sites of the H_2-activated MOF catalyst (measured at room temperature after N_2 purge) provides a quantitative evidence of the defect concentration for ligand-engineered MOF catalysts containing different concentration of defects (see Supplementary Table 1). This experiment also demonstrates the notable concentration of defects already present on non-defective MOF_{L0} originated by thermal treatment at 200 °C under N_2.

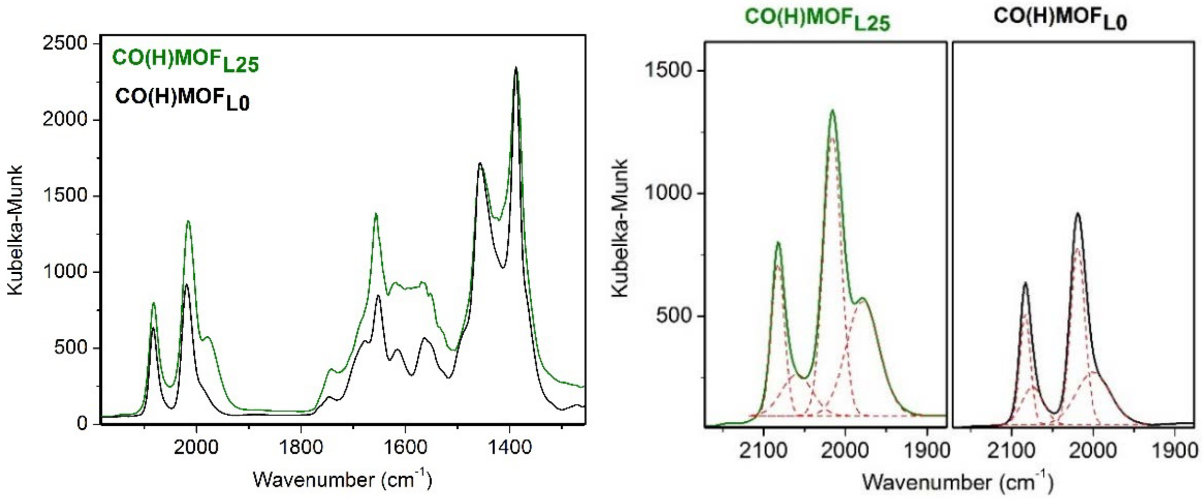

Supplementary Figure 9. DRIFT spectra after CO adsorption on MOF_{L0} and MOF_{L25} (left) and amplified in the Ru–H/Ru–CO region (right) after N_2 treatment at 200 ºC and H_2 treatment at 150 ºC.

Supplementary Table 1. Band position and area of identified species after CO adsorption for MOF_{L0} and MOF_{L25}.

	MOF_{L25}		MOF_{L0}	
	cm^{-1}	Area	cm^{-1}	Area
Ru^{+2}–CO	2082	12394	2083	7420
Ru^{+2}–H	2059	7892	2074	5066
$Ru^{+\delta}$–CO	2015	31299	2018	16055
$Ru^{+\delta}$–H	1978	23867	1998	11272

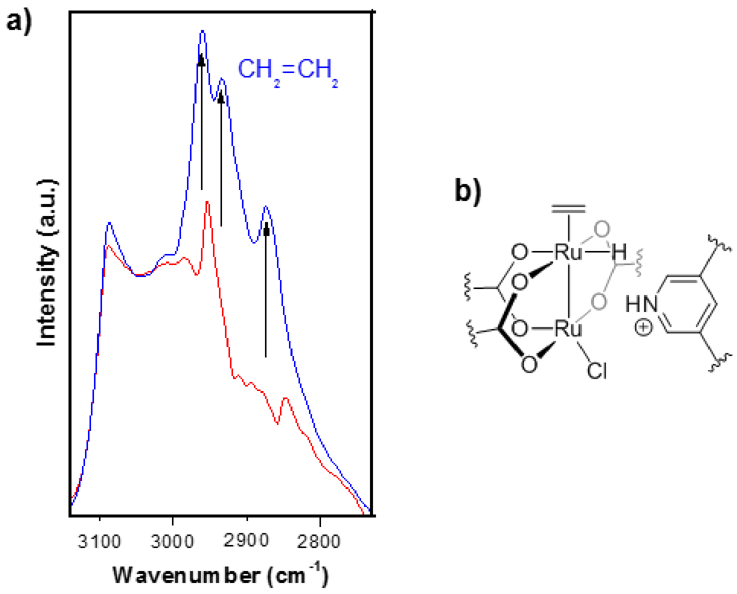

Supplementary Figure 10. a) IR spectra for H_2-activated MOF_{L25} before (red) and after (blue) ethylene adsorption at 50 ºC in the cell. IR measurements carried out in N_2 after ethylene purging for 10 min at 50 ºC. b) Schematic drawing of ligand-engineered MOF after ethylene adsorption.

Supplementary Note 8. Dimerization reactivity of ligand-engineered MOF samples.

As shown in Supplementary Figure 11, dimerization rates per catalyst mass increase as a function of defect incorporation through pyridine ligand-engineering after N_2-treatment at 200 ºC and H_2-treatment at 150 ºC. Highest rates are achieved for 25% ligand-engineered samples, but rate values decline as additional defects are incorporated (50%), indicating the partial structural degradation and microporosity drop above 25% ligand-incorporation (as shown by N_2 sorption isotherms in Supplementary Figure 2).

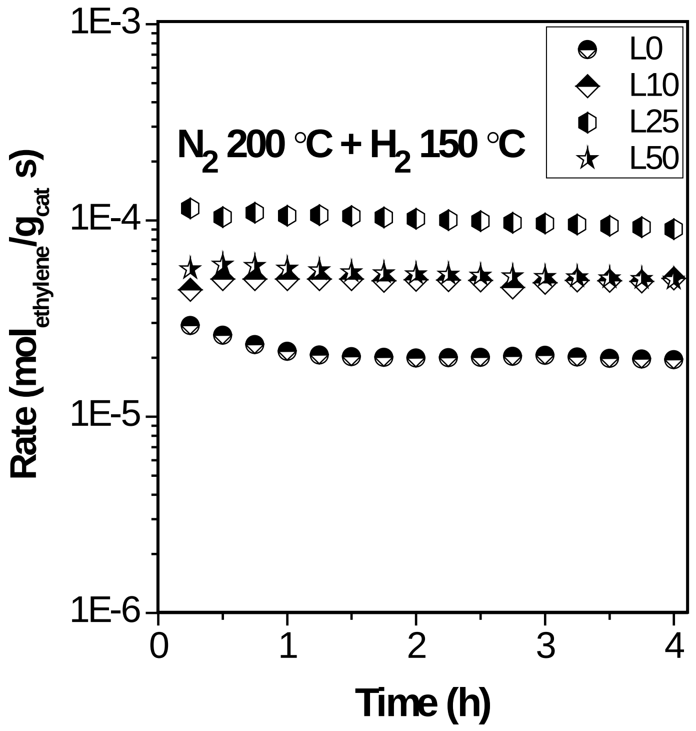

Supplementary Figure 11. Ethylene dimerization reactivity per catalyst mass at 4.2 MPa ethylene and 50 ºC vs time-on-stream for ligand-engineered defects after N_2 treatment at 200 ºC and H_2 treatment at 150 ºC.

Supplementary Note 9. Thermogravimetric analysis (TGA) in N_2 of ligand-engineered MOF samples.

As shown in Figure 3 in the main text, TGA analysis was used to calculate the concentration of defects. In order to calculate the concentration of the thermal-engineered defects, each temperature step was hold for 1 h to reach the equilibrium.

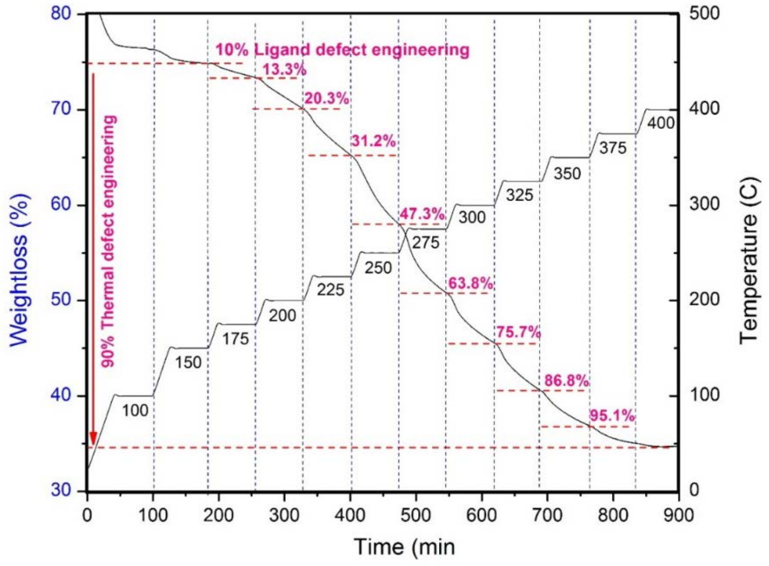

Supplementary Figure 12. TGA in N_2 for MOF_{L10}.

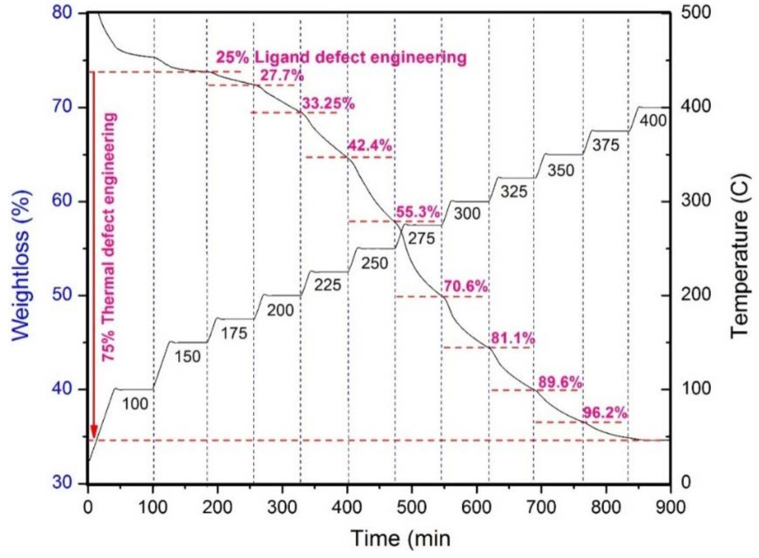

Supplementary Figure 13. TGA in N_2 for MOF_{L25}.

Supplementary Note 10. Thermogravimetric analysis (TGA) + Mass spectrometry (MS) analysis.

TGA-MS analysis combined with TPR-MS analysis was performed for MOF catalysts containing different ligand-engineered defects (MOF_{L0}, MOF_{L10} and MOF_{L25}) under the activation conditions (N_2 treatment at 200 ºC and H_2 treatment at 150 ºC). This experiment evidences the release of H_2O, CO_2, CO, CH_4 and HCl during the N_2-activation at 200 °C, which justifies the presence of CO adsorbed after this treatment. Only H_2O is released during the H_2-treatment at 150 °C. The consumption of H_2 during this treatment is also indicated in the following figures, thus indicating that MOF catalysts containing thermal-engineered defects consume more H_2 during the H_2-treatment to form H_2O from O adsorbed species which may be originated from the CO_2 cleavage.

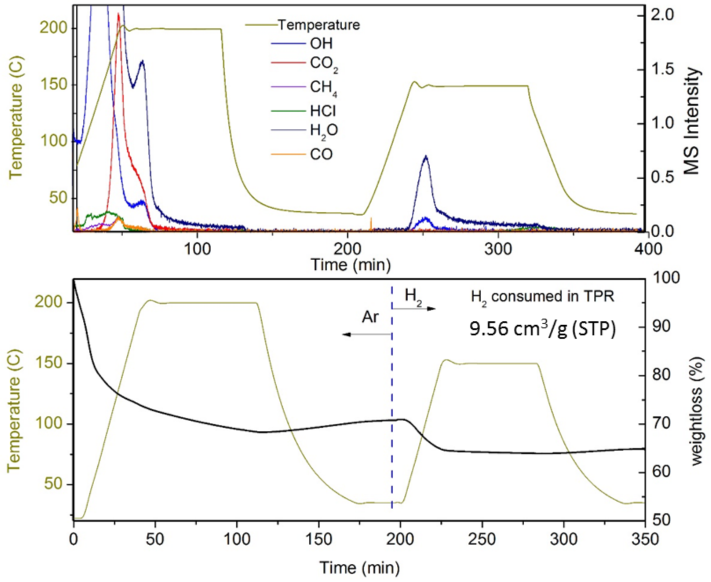

Supplementary Figure 14. TPR-TGA-MS for MOF_{L10}.

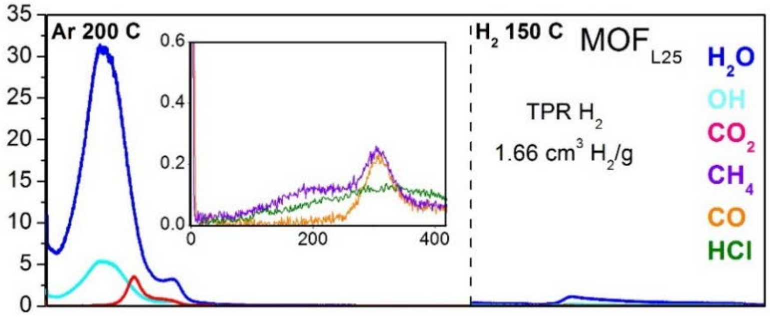

Supplementary Figure 15. TPR-TGA-MS for MOF_{L25}.

Supplementary Note 11. *In situ* IR monitoring of thermal-engineering of defects in MOF_{L0}.

As shown Supplementary Figure 16, in situ IR monitoring of the thermal-engineering of defects in MOF_{L0} shows the evolution of the carboxylates linking Ru paddle-wheels and phenyl rings as well as adsorbed CO and hydrides at each temperature (measured under N_2). More details about the mechanism for thermal engineering of defects on MOF catalysts is shown in Supplementary Figure 16.

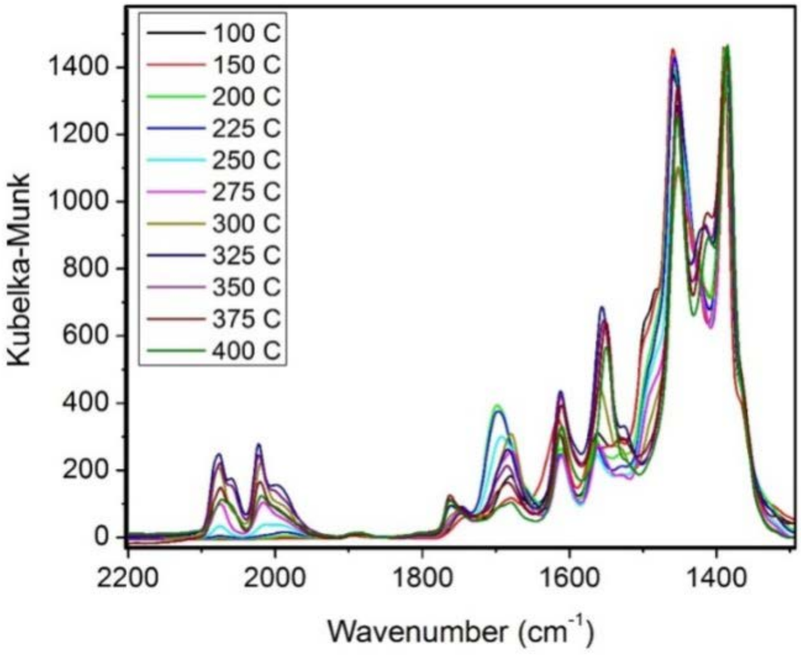

Supplementary Figure 16. *In-situ* IR spectra of MOF_{L0} sample at increasing temperatures under N_2.

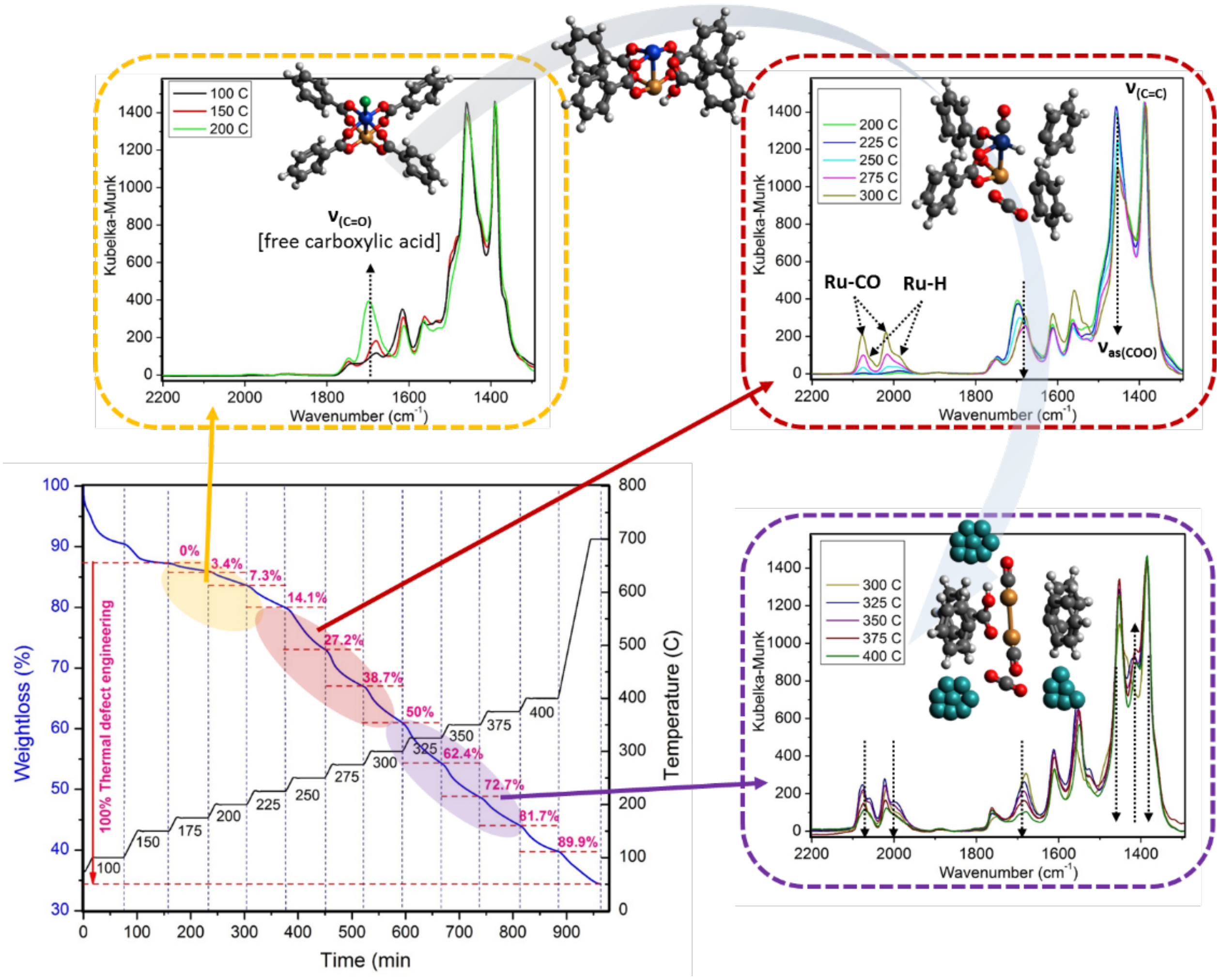

Supplementary Figure 17. Correlation between TGA analysis and *in situ* IR spectra at corresponding temperature ranges for thermal engineering of defects on MOF_{L0}.

Supplementary Table 2. Elemental analysis of thermally-treated MOF_{L0}.

Temperature (°C)	% C	% H	% Cl
**150**	30.17	1.57	6.21
**200**	26.46	1.81	1.62
**300**	23.03	1.64	1.82
**400**	18.97	0.8	0.44

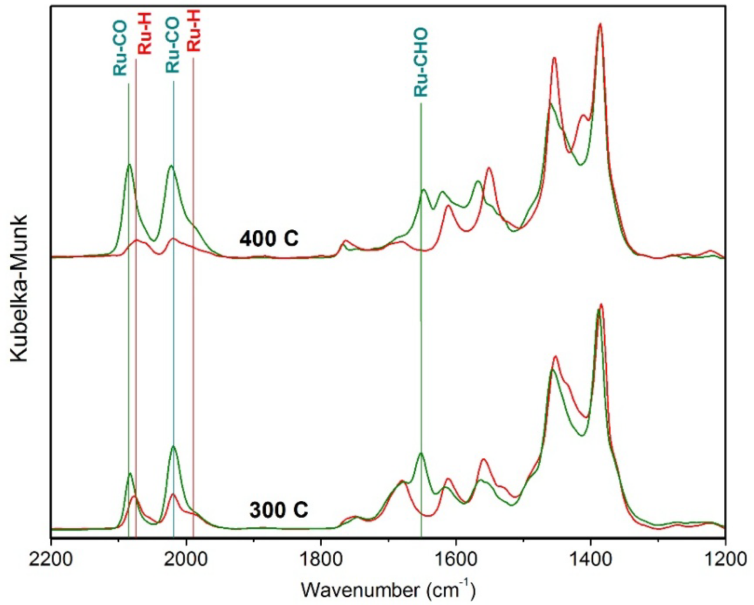

Supplementary Figure 18. N_2 treated (red) and CO adsorbed (green) comparison between MOF_{L0} treated at 300 ºC and 400 ºC in N_2.

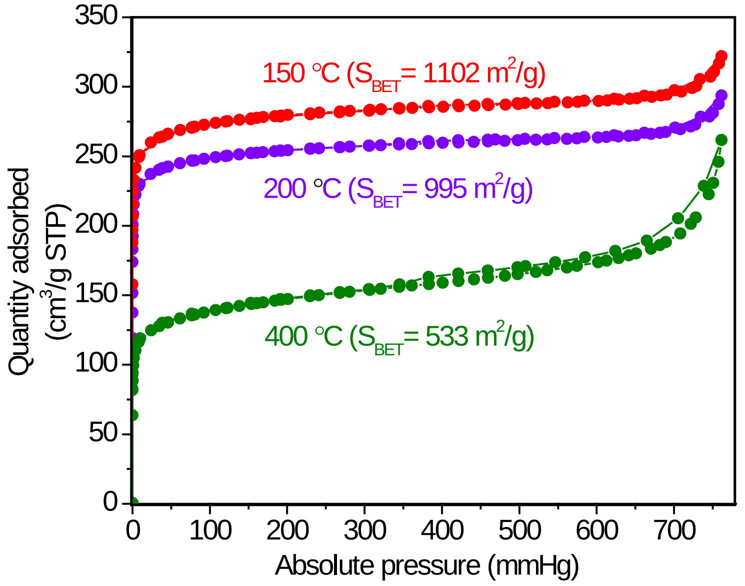

Supplementary Figure 19. N_2 sorption isotherms for MOF_{L0} as a function of thermal treatment in N_2.

Supplementary Note 12. XPS analysis for thermal-engineered defective MOF catalysts.

XPS analysis of the thermal-engineered defective MOF catalysts evidences the advanced reduction of Ru^{+3} to Ru^{+2}/$Ru^{+\delta}$ species at 200 °C and further reduction to Ru^{0} at 400 °C, as seen in Supplementary Figure 20. XPS signals between 292 and 280 eV were more difficult to assign due to the overlapping of Ru(3d) and C(1s) species, as resumed in Supplementary Table 3. Peaks attributed to Cl species are also reduced at 200 °C.

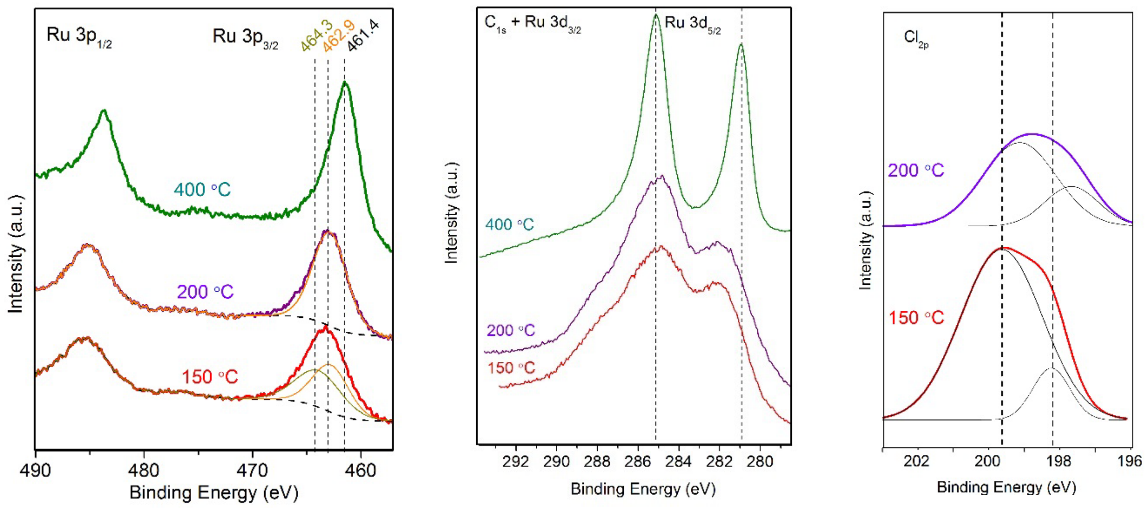

Supplementary Figure 20a. XPS analysis of thermal-engineered MOF catalysts: (left) Ru(3p) (490-455 eV), (middle) Ru_{3d} + C_{1s} (292-280 eV) and (right) Cl_{2p} (203-196 eV).

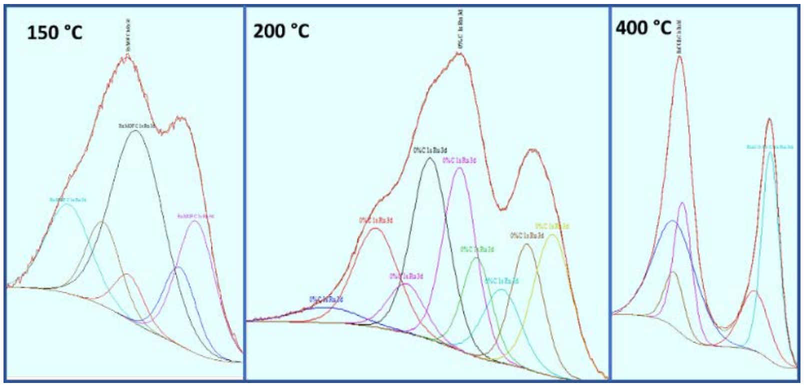

Supplementary Figure 20b. XPS deconvolution plots of Ru(3d) and C(1s) between 292 and 280 eV.)

Supplementary Table 3. % for each species after XPS deconvolution analysis of Ru(3d) and C(1s).

species	Reference[1]	MOF_{L0}(150°C)[a]	(%)[b]	(H)MOF_{L0}(200°C)[a]	%[b]	(H)MOF_{L0}(400°C)[a]	%[b]
**C** [d]	288.5, 285.0	288.5, 285.0	-	288.2, 285.0		285.0	-
**Ru^{+3}**	286.7, 282.5	286.7,282.8	49.2	287.0, 282.9	12.5	-	-
**Ru^{+2}**	285.7, 281.5	285.5,281.9	50.8	286.1, 282.3	37.5	-	-
**$Ru^{+\delta}$**	284.2, 280.6	-	-	284.3, 281.4	50.	285.1, 281.2	31.8
**Ru^{0}**		-	-	-	-	284.8, 280.5	68.2

[a] Binding Energy (eV). [b] Abundance calculated from Ru $3d_{5/2}$ region. Ru $3d_{5/2}$ and $3d_{3/2}$ region for Ru^{+3}, Ru^{+2}, $Ru^{+\delta}$ and Ru^{0}. C_{1s} peak at 285.0 and carboxylate-related C_{1s} peak at 288.5.

Supplementary Note 13. Ethylene dimerization rates for thermal-engineered MOF samples.

Supplementary Figure 21 shows dimerization rates (per catalyst mass) at 50 ºC for MOF_{L0} samples thermally-treated in N_2. Highest rates are achieved for samples containing highest available Ru–H species, as evidenced from IR spectra in Figure 3. MOF_{L0} samples thermally-treated above 300 ºC show much lower rates due to full Ru atom reduction in the presence of excess framework decarboxylation and the slight deactivation due to enhanced at structural collapse.

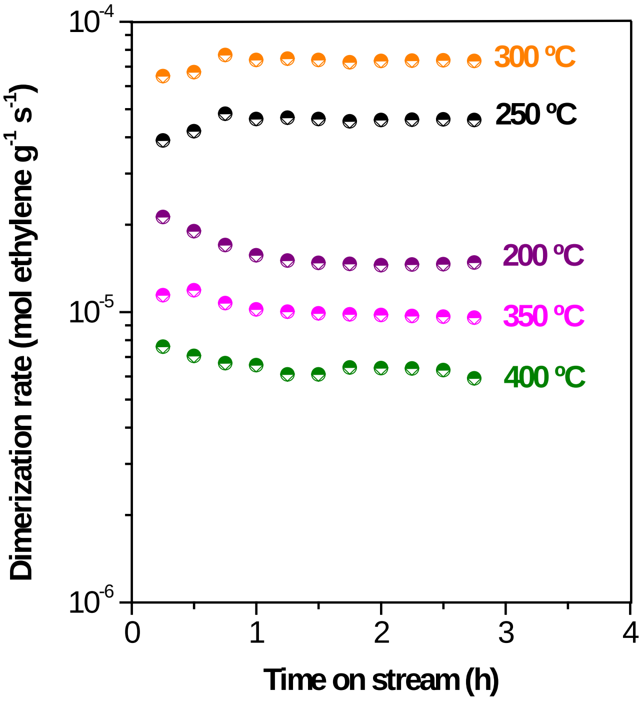

Supplementary Figure 21. Ethylene dimerization rates (per catalyst mass) for MOF_{L0} as a function of thermal treatment in N_2. Reaction conditions at 50 ºC and 4.2 MPa ethylene.

Supplementary Note 14. Comparison of ethylene dimerization turnovers numbers for MOF_{L25} and reported Ni-based MOFs.

Supplementary Table 4 shows dimerization TOFs for reported Ni-MOFs and this study. Most Ni-MOFs use co-catalysts and solvents in reaction, and lower TOFs are obtained under gas-conditions. Moreover, Ni-MOFs deactivate time-on-stream and do not show regeneration methods. This study shows comparable TOF values using Ru-MOFs in the presence of intrapore liquids and the absence of co-catalysts and solvents.

Supplementary Table 4. MOF-catalyzed ethene dimerization turnovers, stability and 1-butene selectivities.

Support	Activator /metal	Phase	Temperature (°C)	TOF (per metal, M) ($mol_{butene}\ mol_M^{-1}\ h^{-1}$)	Butene selectivity (% mol)	Mean life $1/k_d$ (h)[a]	TON	Ref.
Ni-(Fe)MIL-101	70 (Al)	Liquid (batch)	10	10400	94	-	-	2
Ni-MFU4*l*	500 (Al)	Liquid (batch)	25	41500	94	-	-	3
$Zr_6O_4(OH)_4(bpydc)_6$	100 (Al)	Liquid (batch)	55	36000	73	-	-	4
$(bpy)Ni^{II}$	70 (Al)	Gas/Liquid (flow)	24	1570	97	12	16000 (after 12 h)	5
Ni-AIM	DMF/HCl	Gas (flow)	45	252	46	-	-	6
Ni-Facac-AIM-NU-1000	Et_2AlCl	Gas (flow)	45	16	99	10	160	7
RuDEMOF	Et_2AlCl	Heptane	80	4	99	-	-	8
(Ru)HKUST-1	0	Gas (flow)	60	200[b]	99	>120	13000 (after 120 h)	This

[a] k_d: first order deactivation constant.

[b] Turnovers measured per Ru active, as determined from IR and DFT

Supplementary Note 15. Long-term ethylene dimerization rates MOF.

Supplementary Figure S22 shows ethylene dimerization rates for MOF_{L10} during 120 h time on stream and in the presence of intrapore liquid-ethylene. The MOF_{L10} catalyst shows remarkable dimerization rates, attributed to intrapore liquids that solvate specific desorption intermediate before bound oligomer growth.

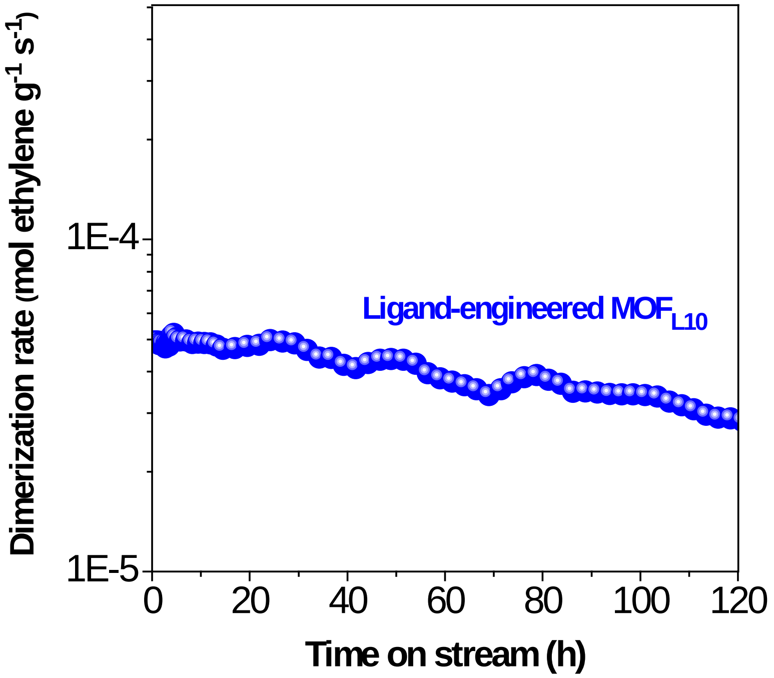

Supplementary Figure 22. Ethylene dimerization rates per catalyst mass at 4.2 MPa ethylene and 50 ºC *vs.* time-on-stream for ligand-engineered MOF_{L10} after N_2 treatment at 200 ºC and H_2 treatment at 150 ºC.

Supplementary Note 16. Ethylene relative pressure at operating dimerization conditions (4.2 MPa).

Supplementary Figure 23 shows ethylene relative saturation pressure (P/P_{sat}, taking 4.2 MPa as reference pressure) using ethylene saturation pressure (P_{sat}) at a given temperature. As temperature increases, P/P_{sat} decreases. Capillary condensation effects for Ru-based MOF catalysts (1.5 nm pore aperture) at 50 ºC were observed above P/P_{sat} of 0.4. This suggests that narrower pores in MOFs (0.5 nm) condensing ethylene at P/P_{sat} of 0.15 could achieve ethylene condensation at higher temperatures. According to Supplementary Figure 23, narrow pores could condense ethylene at P/P_{sat} ~ 0.15 at 150 ºC, achieving both kinetic and stability benefits.

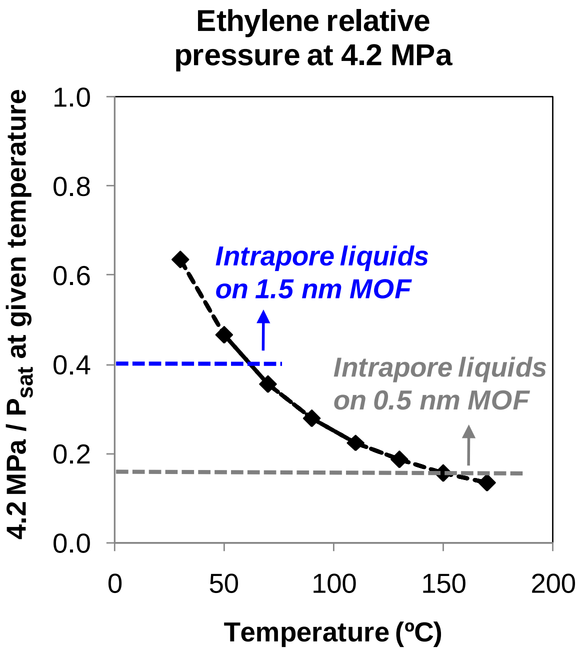

Supplementary Figure 23. Ethylene relative saturation pressure (4.2 MPa / P_{sat}) as a function of temperature. Blue line represents the minimum P/P_{sat} value to have intrapore liquids in 1.5 nm MOF and grey line represents the minimum P/P_{sat} value to have intrapore liquids in 0.5 nm MOF catalysts.

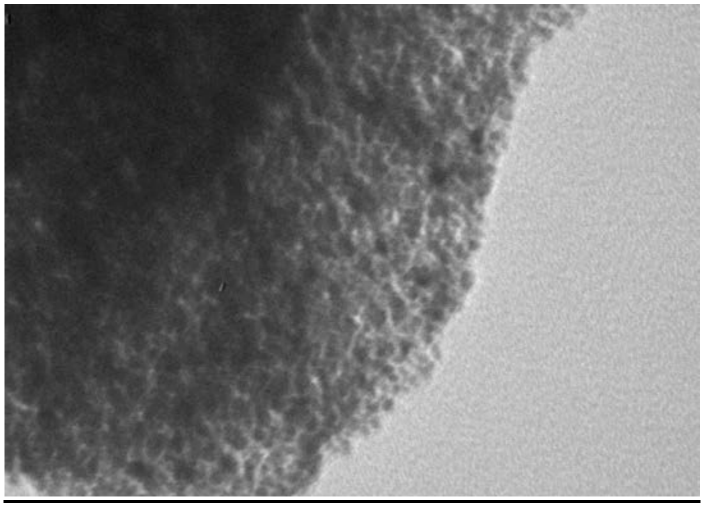

Supplementary Figure 24. TEM analysis of Ru nanoparticles on a carbonaceous matrix after heating at 500 ºC in N_2.

Supplementary Note 17. Solid-state ^{13}C NMR data.

Supplementary Supplementary Figure 25 shows the ^{13}C NMR CP-MAS analysis for pristine (Ru)-HKUST-1 material, which is compared to the spectra of the MOF ligand. The paramagnetic nature of such Ru-based MOF structures, however, leads to a very broad band for phenyl carbons (130 ppm) and very noisy or absent carboxylic carbons, in contrast to the sharp ^{13}C band obtained for the trimesic acid (MOF ligand).

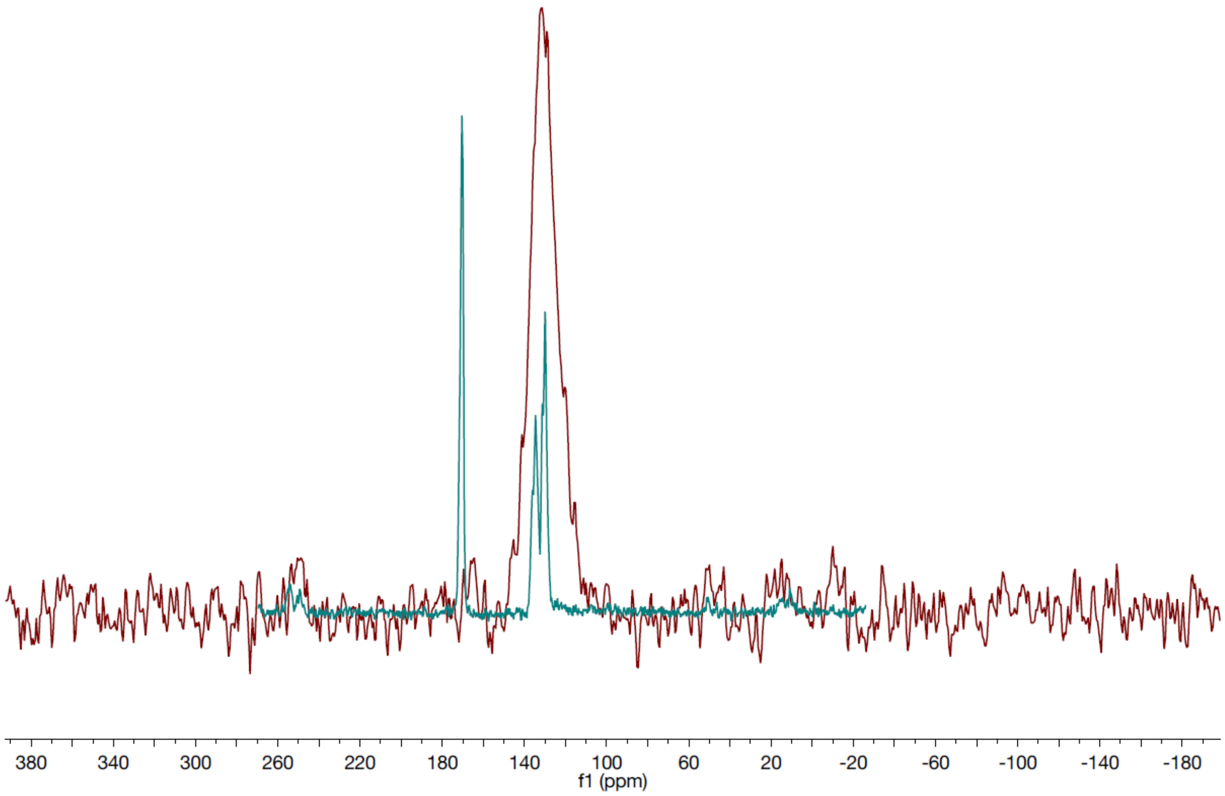

Supplementary Figure 25. ^{13}C NMR CP-MS spectra of pristine (Ru)-HKUST-1 (red) and spectra of benzene-1,3,5-tricarboxylate MOF ligand (green).

Supplementary Note 18. Computational details.

All inputs, outputs, and final structures can be found in the ioChem-BD repository.[9,10]

Periodic methods details. Periodic calculations were performed at the DFT level using the Vienna Ab-initio Simulation Package (VASP).[11,12] The PBE functional[13] was used together with Grimme's D2 dispersion scheme[14] and modified parameters for transition metals.[15] This functional performs well for structural parameters of MOFs.[16] Core electrons were described by projector augmented wave (PAW)[17] and valence electrons in plane waves with a kinetic energy cutoff of 450 eV. Cell optimization of the pristine MOF was performed with a cutoff of 600 eV. The Brillouin zone was sampled at the Γ-point through the Monkhorst–Pack method.[18] Transition states (TSs) were located with the climbing image nudged elastic band method.[19] The assessment of the minima and transition states was performed by diagonalizing the numerical Hessian matrix obtained by ± 0.015 Å displacements. Only selected atoms were allowed to move during frequency calculations. A benchmark calculation was performed at HSE03 level[20,21] including 13% exact exchange.[22]

Cluster methods details. Cluster calculations were performed at the DFT level using Gaussian09.[23] Ru atoms were described with a SDD pseudopotential and its associated basis set;[24] H, C, O, and Cl atoms were described with the 6-31G** basis set.[25,26] Diffuse functions were added to O and Cl atoms.[27] The following density functionals were tested: M06-L,[28,29] which can capture the multiconfigurational character of metallic nodes,[30] PBE-D3,[13,31] and B3LYP-D3,[31–34] commonly used for paddlewheel cluster models[35,36] and molecular complexes.[37,38] We cropped one bimetallic node with eight linkers from the periodic structure; the carboxylic groups of the linkers were capped with hydrogen atoms and the four *p*-carbon atoms were kept fixed during geometry optimization to mimic the mechanic constraint of the framework.

Supplementary Note 19. Periodic models for pristine and defective MOFs.

The initial cubic unit cell of the Ru-MOF (a = 26.634 Å) containing 24 nodes was taken from literature;[39] missing H and Cl atoms were added according to previous studies (Supplementary Figure 26a).[35] Notice that although Cl would be randomly oriented in the material, the use of periodic boundary conditions (PBC) assumes the same local structure for all the nodes. We introduced the ligand-based defect by modifying one 1,3,5-benzenetricarboxylate of one node to pyridine-3,5-dicarboxylate (Supplementary Figure 26b). Similarly, we introduced the thermal-based defect by changing one carboxylic group of one node to hydrogen and removing one Cl to mimic the thermal decarboxylation (Supplementary Figure 26c). The large extension of the unit cell precludes the spurious high concentration of defects due to PBC.

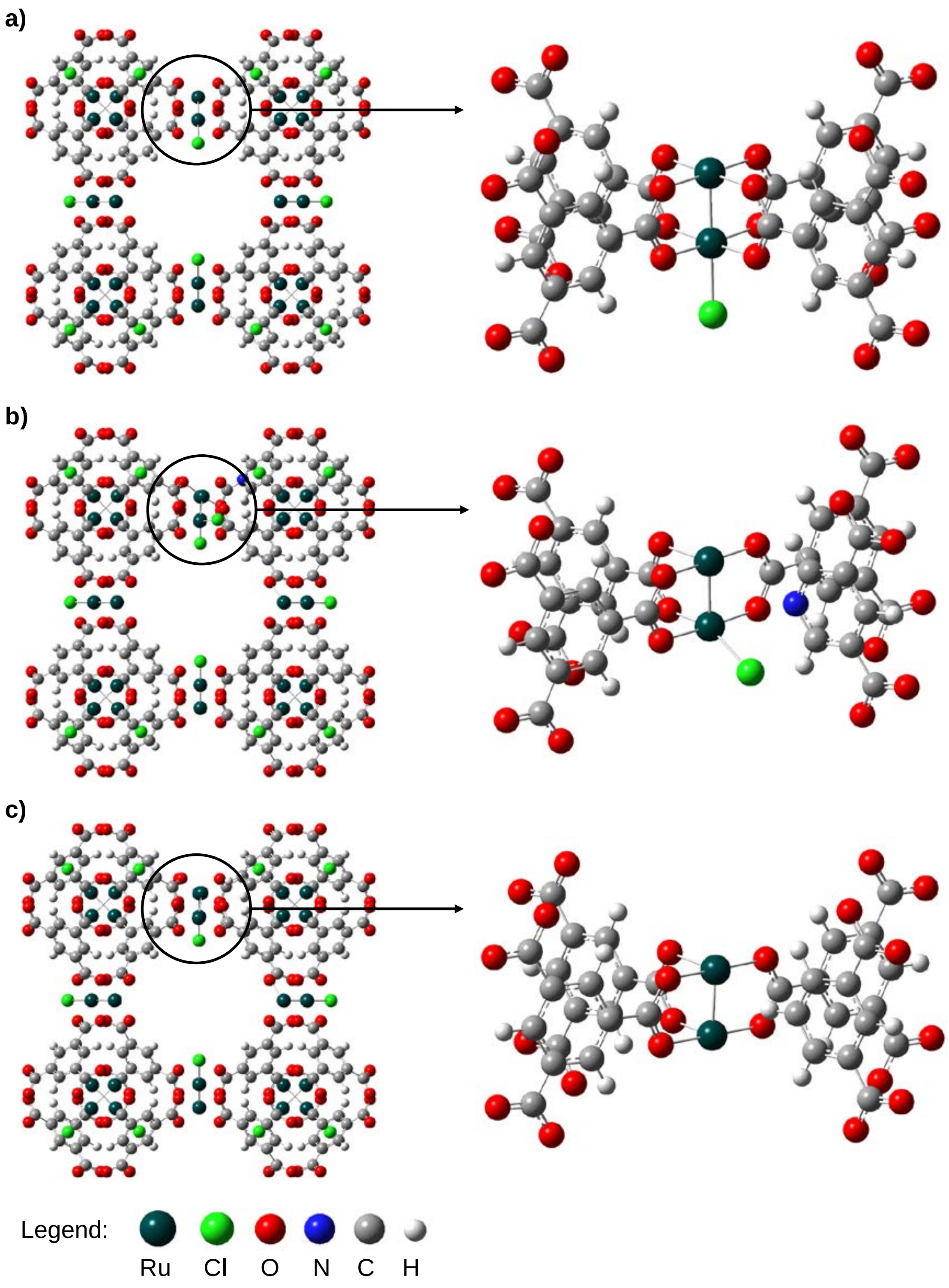

Supplementary Figure 26. Optimized structures of (a) pristine, (b) ligand-based, and (c) thermal-based defective MOFs.

Supplementary Note 20. Benchmark calculations.

In this section we consider quartet and doublet spin states for the pristine bimetallic MOF node using cluster and periodic models (Supplementary Table 5). For cluster calculations we use M06-L, PBE-D3, and B3LYP-D3 density functionals. We found the quartet as the most stable spin state. Despite having Ru atoms with different chemical environments, they show similar spin densities of *ca.*1.2 and 1.5 for all three density functionals. Periodic calculations at PBE-D2 level are in agreement with cluster calculations. Moreover, a single-point calculation using the hybrid functional HSE03-13[20–22] provided the same result, which further validates the use of the more computationally affordable PBE protocol for periodic calculations.

Supplementary Table 5. Spin densities and relative spin state energies of bimetallic nodes on pristine MOF at different levels of theory.

Method	M06-L		PBE-D3		B3LYP-D3		Periodic PBE-D2	Periodic HSE-13
Spin state	Quartet[a]	Doublet	Quartet	Doublet	Quartet	Doublet	Quartet[c]	Quartet[c]
ρ(Ru–Cl)	1.24	0.52	1.26	0.47	1.19	0.53	~1.25	~1.25
ρ(Ru–vacancy)	1.47	0.41	1.39	0.44	1.48	0.40	~1.33	~1.40
Energy / eV	0.00	0.52[b]	0.00	0.43[b]	0.00	0.63	-	-

[a] The sextet spin state is found at 1.41 eV. [b] Yamaguchi-corrected value.[40,41] Quartet spin state per node, i.e., 72 unpaired electrons overall.

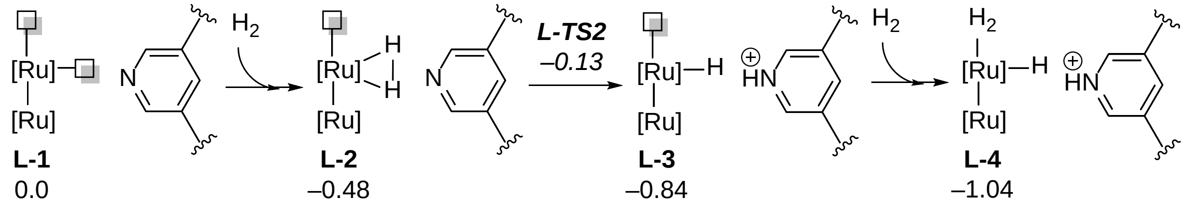

Supplementary Figure 27. Computed H_2 heterolytic cleavage promoted by a frustrated Lewis pair in ligand-engineered (**L**) defective nodes. Potential energies referred to **L-1** and gas-phase H_2 (in eV).

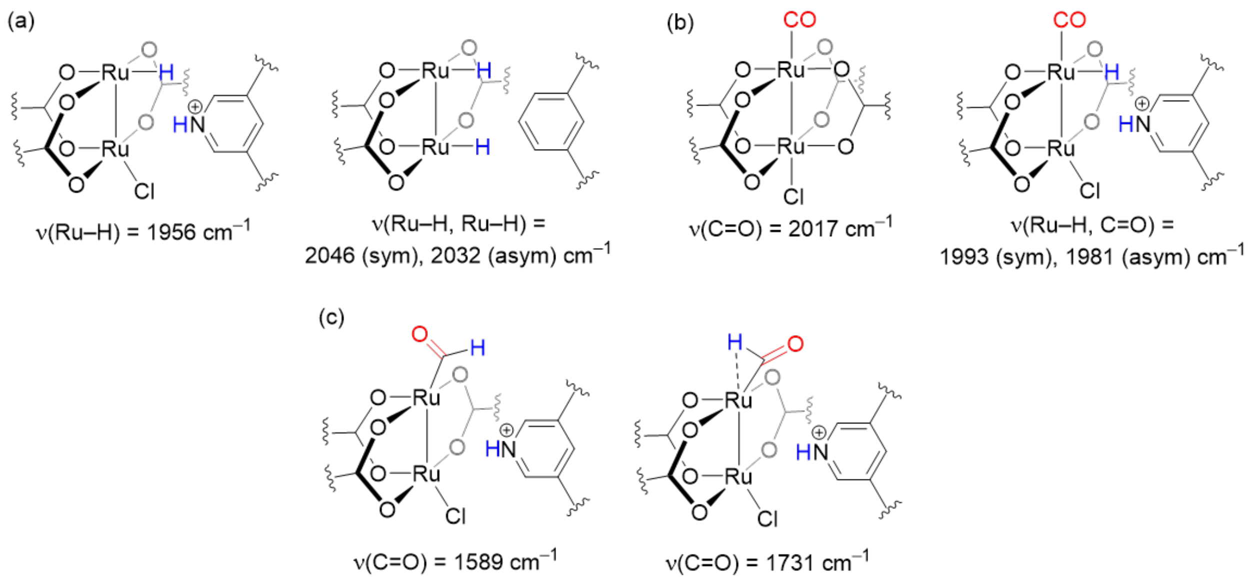

Supplementary Figure 28. Computed frequencies of (a) hydride, (b) CO, and (c) formyl ligands on MOF nodes.

Supplementary Note 21. Influence of spin state.

The reaction mechanism reported in the main text (Figure 4c) was calculated considering 73 unpaired electrons for ligand-engineered (**L**) species. Here, we compute the key steps of the mechanism considering 71 unpaired electrons (Supplementary Figure 29). Although alkyl intermediates are more stable in a lower spin state, the energy profile is essentially the same for both spin surfaces.

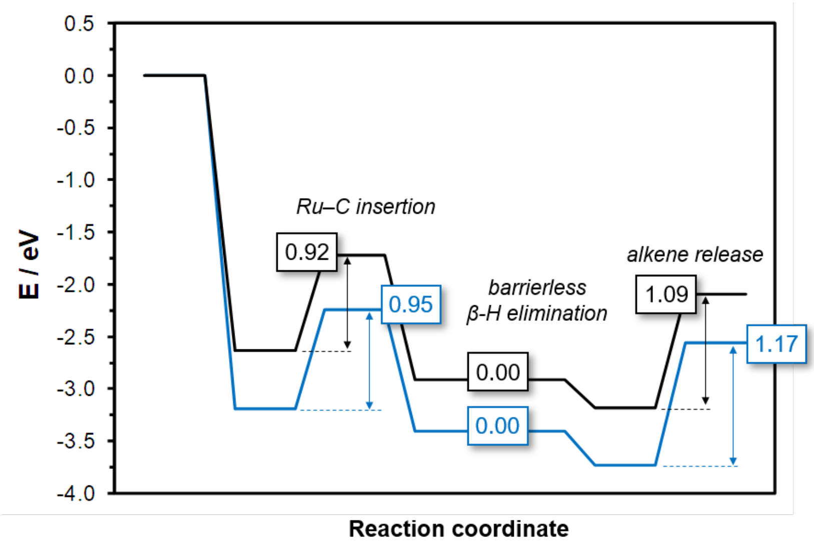

Supplementary Figure 29. Computed reaction energy profile for insertion and alkene release steps in high (black) and low (blue) spin surfaces.

Supplementary Note 22. Influence of secondary metal on alkene desorption.

The original MOF node contains two Ru atoms, but only one is actively participating in the reaction (Figure 4). To test the influence of the second Ru, we prepared a node model by removing the $[Ru^{II}Cl]^{+}$ unit and adding one proton to one carboxylate linkers to balance charge. The alkene release step *via* the original defective node (Supplementary Figure 30a) is 0.65 eV more favorable than that *via* the monometallic model (Supplementary Figure 30b). We suggest that the secondary Ru atom strongly influences the coordination shell of the reactive Ru by promoting ligand desorption. These results support the need of bimetallic nodes to enhance selectivity.

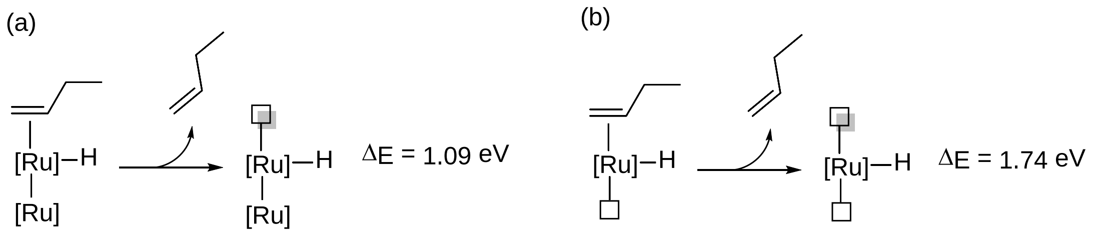

Supplementary Figure 30. Alkene desorption energies for (a) bimetallic ligand-based defective node and (b) monometallic model.

Supplementary References

1. Agirrezabal-Telleria, I., Iglesia, E. Stabilization of active, selective, and regenerable Ni-based dimerization catalysts by condensation of ethene within ordered mesopores. *J. Catal.* **352**, 505–514 (2017).
2. Canivet, J., Aguado S., Schuurman Y. Farrusseng D., MOF-supported selective ethylene dimerization single-site catalysts through one-pot postsynthetic modification, *J. Am. Chem. Soc*. **135**, 4195−4198 (2013).
3. Metzger, E. D., Brozek, C. K., Comito, R. J., Dincă, M., Selective dimerization of ethylene to 1-butene with a porous catalyst. *ACS Cent. Sci*. **2**, 148–153 (2016).
4. Gonzalez, M. I., Oktawiec, J., Long, J. R., Ethylene oligomerization in metal–organic frameworks bearing nickel(II) 2,2′-bipyridine complexes, *Faraday Discuss.*, **201**, 351–367 (2017).
5. Madrahimov, S. T., Gallagher, J. R., Zhang, G., Meinhart, Z., Garibay, S. J., Delferro, M., Miller, J. T., Farha, O. K., Hupp, J. T., Nguyen, S. T., Gas-phase dimerization of ethylene under mild conditions catalyzed by MOF materials containing (bpy)Ni^{II} complexes. *ACS Catal*. **5**, 6713–6718 (2015).
6. Li, Z., Schweitzer, N. M., League, A. B. Bernales, V., Peters, A.W., Getsoian, A. B., Wang, T. C., Miller, J. T., Vjunov, A., Fulton, J. L., Lercher, J. A., Cramer, C. J., Gagliardi, L., Hupp, J. T., Farha, O. K., Sintering-resistant single-site nickel catalyst supported by metal–organic framework, *J. Am. Chem. Soc*. **138**, 1977–1982 (2016).
7. Liu, J., Ye, J., Li, Z., Otake, K., Liao, Y., Peters, A. W., Noh, H., Truhlar, D. G., Gagliardi, L., Cramer, C. J., Farha, O. K., Hupp, J. T., Beyond the active site: tuning the activity and selectivity of a metal−organic framework-supported Ni catalyst for ethylene dimerization. *J. Am. Chem. Soc.* **140**, 11174–11178 (2018).
8. Zhang, W., Kauer, M., Halbherr, O., Epp, K., Guo, P., Gonzalez, M. I., Xiao, D. J., Wiktor, C., LIabrés i Xamena, F.-X., Wöll, C., Wang, Y., Muhler, M., Fischer, R. A. Ruthenium metal–organic frameworks with different defect types: influence on porosity, sorption, and catalytic properties, *Chem. Eur. J.* **22**, 14297–14307 (2016).
9. Alvarez-Moreno, M., De Graaf, C., López, N., Maseras, F., Poblet, J. M., Bo, C. Managing the computational chemistry big data problem: the ioChem-BD platform. *J. Chem. Inf. Model.* **55**, 95–103 (2015).
10. Ortuño, M. A. Database, ioChem-BD DOI: http://dx.doi.org/10.19061/iochem-bd-1-83.
11. Kresse, G., Furthmüller, J. Efficient iterative schemes for ab initio total-energy calculations using a plane-wave basis set. *Phys. Rev. B: Condens. Matter Mater. Phys.* **54**, 11169–11186 (1996).
12. Kresse, G., Furthmüller, J. Efficiency of ab-initio total energy calculations for metals and semiconductors using a plane-wave basis set. *Comput. Mater. Sci.* **6**, 15–50 (1996).

13. Perdew, J. P., Burke, K. &Ernzerhof, M. Generalized gradient approximation made simple. *Phys. Rev. Lett.* **77**, 3865–3868 (1996).

14. Grimme, S. Semiempirical GGA-type density functional constructed with a long-range dispersion correction. *J. Comp. Chem.* **27**, 1787–1799 (2006).

15. Almora-Barrios, N., Carchini, G., Błoński, P.&López, N. Costless derivation of dispersion coefficients for metal surfaces. *J. Chem. Theory Comput.* **10**, 5002–5009 (2014).

16. Nazarian, D., Ganesh, P., Sholl, D. S. Benchmarking density functional theory predictions of framework structures and properties in a chemically diverse test set of metal–organic frameworks. *J. Mater. Chem. A* **3**, 22432–22440 (2015).

17. Kresse, G., Joubert, D. Ultrasoft pseudopotentials to the projector augmented-wave method. *Phys. Rev. B: Condens. Matter Mater. Phys.* **59**, 1758–1775 (1999).

18. Monkhorst, H. J., Pack, J. D. Special points for Brillouin-zone integrations. *Phys. Rev. B* **13**, 5188–5192 (1976).

19. Henkelman, G., Jónsson, H. Improved tangent estimate in the nudged elastic band method for finding minimum energy paths and saddle points. *J. Chem. Phys.***113**, 9978–9985 (2000).

20. Heyd, J., Scuseria, G. E., Ernzerhof, M. Hybrid functionals based on a screened Coulomb potential. *J. Chem. Phys.* **118**, 8207 (2003).

21. Heyd, J., Scuseria, G. E. Assessment and validation of a screened Coulomb hybrid density functional. *J. Chem. Phys.* **120**, 7274 (2004).

22. Hegner, F. S., Herraiz-Cardona, I., Cardenas-Morcoso, D., López, N., Galán-Mascarós, J. R.&Gimenez, S. Cobalt hexacyanoferrate on $BiVO_4$ photoanodes for robust water splitting. *ACS Appl. Mater. Interfaces* **9**, 37671–37681 (2017).

23. Frisch, M. J. et al. Gaussian 09, Revision D.01; Gaussian, Inc.: Wallingford, CT, 2010.

24. Andrae, D., Haeussermann, U., Dolg, M., Stoll, H., Preuss, H. Energy-adjusted ab initio pseudopotentials for the second and third row transition elements. *Theor. Chim. Acta* **77**, 123–141 (1990).

25. Hehre, W. J., Ditchfield, R., Pople, J. A. Self-consistent molecular orbital methods. XII. Further extensions of Gaussian-type basis sets for use in molecular orbital studies of organic molecules. *J. Chem. Phys.* **56**, 2257 (1972).

26. Hariharan, P. C., Pople, J. A. The influence of polarization functions on molecular orbital hydrogenation energies. *Theor. Chim. Acta* **28**, 213–222 (1973).

27. Clark, T., Chandrasekhar, J., Spitznagel, G. W., Schleyer, P. Efficient diffuse function-augmented basis sets for anion calculations. III. The 3-21+G basis set for first- row elements, Li –F. *J. Comput. Chem.***4**, 294–301 (1983).

28. Zhao, Y., Truhlar, D. G. A new local density functional for main-group thermochemistry, transition metal bonding, thermochemical kinetics, and noncovalent interactions. *J. Chem. Phys.* **125**, 194101 (2006).
29. Bernales, V., Ortuño, M. A., Truhlar, D. G., Cramer, C. J., Gagliardi, L. Computational design of functionalized metal–organic framework nodes for catalysis. *ACS Cent. Sci.* **4**, 5–19 (2018).
30. Verma, P., Vogiatzis, K. D., Planas, N., Borycz, J., Xiao, D. J., Long, J. R., Gagliardi, L., Truhlar, D. G. Mechanism of oxidation of ethane to ethanol at iron(IV)–oxo sites in magnesium-diluted Fe_2(dobdc). *J. Am. Chem. Soc.* **137**, 5770–5781 (2015).
31. Grimme, S., Antony, J., Ehrlich, S., Krieg, H. A consistent and accurate ab initio parametrization of density functional dispersion correction (DFT-D) for the 94 elements H-Pu. *J. Chem. Phys.* **132**, 154104 (2010).
32. Becke, A. D. Density-functional exchange-energy approximation with correct asymptotic behavior. *Phys. Rev. A: At., Mol., Opt. Phys.* **38**, 3098 (1988)
33. Lee, C., Yang, W., Parr, R. G. Development of the Colle-Salvetti correlation-energy formula into a functional of the electron density. *Phys. Rev. B: Condens. Matter Mater. Phys.* **37**, 785 (1988).
34. Stephens, P. J., Devlin, F. J., Chabalowski, C. F., Frisch, M. J. Ab initio calculation of vibrational absorption and circular dichroism spectra using density functional force fields. *J. Phys. Chem.* **98**, 11623–11627 (1994).
35. Noei, H., Kozachuk, O., Amirjalayer, S., Bureekaew, S., Kauer, M., Schmid, R., Marler, B., Muhler, M., Fischer, R. A., Wang, Y. CO adsorption on a mixed-valence ruthenium metal–organic framework studied by UHV-FTIR spectroscopy and DFT calculations. *J. Phys. Chem. C* **117**, 5658–5666 (2013).
36. Supronowicz, B., Mavrandonakis, A., Heine, T. Interaction of small gases with the unsaturated metal centers of the HKUST-1 metal organic framework. *J. Phys. Chem. C* **17**, 14570–14578 (2013).
37. Fritsch, N., Wick, C. R., Waidmann, T., Dral, P. O., Tucher, J., Heinemann, F. W., Shubina, T. E., Clark, T., Burzlaff, N. Multiply bonded metal(II) acetate (rhodium, ruthenium, and molybdenum) complexes with the *trans*-1,2-bis(N-methylimidazol-2-yl)ethylene ligand. *Inorg. Chem.* **53**, 12305–12314 (2014).
38. Brown, T. R., Dolinar, B. S., Hillard, E. A., Clérac, R., Berry, J. F. Electronic structure of Ru_2(II,II) oxypyridinates: synthetic, structural, and theoretical insights into axial ligand binding. *Inorg. Chem.* **54**, 8571–8589 (2015).
39. Kozachuk, O.; Yusenko, K.; Noei, H.; Wang, Y.; Walleck, S.; Glaser, T.; Fischer, R. A. Solvothermal growth of a ruthenium metal–organic framework featuring HKUST-1 structure type as thin films on oxide surfaces. *Chem. Commun.***47**, 8509–8511 (2011).

40. Yamaguchi, K., Jensen, F.; Dorigo, A., Houk, K. N. A spin correction procedure for unrestricted Hartree-Fock and Møller-Plesset wave functions for singlet diradicals and polyradicals. *Chem. Phys. Lett.* **149**, 537–542 (1988).
41. Soda, T., Kitagawa, Y., Onishi, T., Takano, Y., Shigeta, Y., Nagao, H., Yoshioka, Y., Yamaguchi, K. Ab initio computations of effective exchange integrals for H–H, H–He–H and Mn_2O_2 complex: comparison of broken-symmetry approaches. *Chem. Phys. Lett.* **319**, 223–230 (2000).
